# Effectiveness of mesenchymal stem cells for treating patients with knee osteoarthritis: a meta-analysis toward the establishment of effective regenerative rehabilitation

**DOI:** 10.1038/s41536-018-0041-8

**Published:** 2018-09-17

**Authors:** Hirotaka Iijima, Takuya Isho, Hiroshi Kuroki, Masaki Takahashi, Tomoki Aoyama

**Affiliations:** 1 Department of System Design Engineering, Keio University, Yokohama, Japan; 2 Department of Physical Therapy, Human Health Sciences, Graduate School of Medicine, Kyoto University, Kyoto, Japan; 3 Japan Society for the Promotion of Science, Tokyo, Japan; 4 Rehabilitation Center, Fujioka General Hospital, Gunma, Japan

## Abstract

This systematic review with a meta-analysis aimed to summarize the current evidence of the effectiveness of mesenchymal stem cell (MSC) treatment for knee osteoarthritis (OA) and to examine whether rehabilitation is an effect modifier of the effect estimate of MSC treatment. A literature search yielded 659 studies, of which 35 studies met the inclusion criteria (*n* = 2385 patients; mean age: 36.0–74.5 years). The meta-analysis results suggested that MSC treatment through intra-articular injection or arthroscopic implantation significantly improved knee pain (standardized mean difference [SMD]: −1.45, 95% confidence interval [CI]: −1.94, −0.96), self-reported physical function (SMD: 1.50, 95% CI: 1.09, 1.92), and cartilage quality (SMD: −1.99; 95% CI: −3.51, −0.47). However, the MSC treatment efficacy on cartilage volume was limited (SMD: 0.49; 95% CI: −0.19, 1.16). Minor adverse events (knee pain or swelling) were reported with a wide-ranging prevalence of 2–60%; however, no severe adverse events occurred. The evidence for these outcomes was “very low” to “low” according to the Grades of Recommendation, Assessment, Development and Evaluation system because of the poor study design, high risk of bias, large heterogeneity, and wide 95% CI of the effects estimate. Performing rehabilitation was significantly associated with better SMD for self-reported physical function (regression coefficient: 0.881, 95% CI: 0.049, 1.712; *P* = 0.039). We suggest that more high quality randomized controlled trials with consideration of the potential rehabilitation-driven clinical benefit would be needed to facilitate the foundation of effective MSC treatment and regenerative rehabilitation for patients with knee OA.

## Introduction

Osteoarthritis (OA) is the most common form of arthritis.^1^ OA ultimately results in cartilage degeneration, chronic knee pain, and disability. In 2010, knee OA was the 11th leading cause of disability worldwide, with increasing incidence over the last 2 decades.^2^ Current treatments have little impact on the progressive degeneration of articular cartilage; therefore, developing effective and financially viable disease-modifying therapies is a critical medical priority.

Mesenchymal stem cells (MSCs) have emerged as a cell type with great potential for cell-based articular cartilage repair in patients with knee OA.^3^ Clinical trials that investigate the effects of MSC treatments in patients with knee OA have recently begun emerging,^4^ and results of clinical studies are continuously reported.^5,6^ Several meta-analyses summarize the effects of MSC treatment in patients with knee OA;^7–10^ these studies contribute to the establishment of effective cell-based therapies for degenerative cartilage disease. However, some of these systematic reviews included patients with focal cartilage lesions^8–10^ or focused on pain and physical function as treatment outcomes,^7,9,10^ with a large heterogeneity and lack of evaluation of bias risk.^7–9^ As knee pain would be discordant with articular cartilage status, understanding the effects of MSC treatment against OA joint degeneration and exploring the mechanisms underlying symptom-modifying MSC treatment are important. In addition, confidence in the effects estimate from meta-analysis depends on the quality of the included studies and analytical process,^11^ as the former can be evaluated using the Grades of Recommendation, Assessment, Development and Evaluation (GRADE) approach.^12^ However, no meta-analysis has examined the effects of MSCs on knee OA considering the GRADE approach.

Physical factors such as rehabilitation programs are potential effect modifiers that were not well addressed in previous meta-analyses.^7–10^ Physical factors regulate MSC differentiation and tissue development, pointing to a potential therapeutic strategy for enhancing the MSCs injected or implanted into the knee joint,^13,14^ such as the recently proposed new field “regenerative rehabilitation”.^15^ Regenerative rehabilitation is defined as the integration of principles and approaches from the fields of rehabilitation science and regenerative medicine.^16^ The efficacy of regenerative medicine may be enhanced when coupled with mechanical input. Weight-bearing might influence the structural outcome in the postoperative phase of autologous chondrocyte implantation in adults with cartilage defects.^17,18^ Thus, further investigation of the effects of MSC treatment in patients with knee OA and the potential role of rehabilitation (i.e., regenerative rehabilitation) as an effect modifier would be of interest.

Potential adverse effects have a considerable impact on patient adherence to MSC treatment. To achieve a balanced perspective, a systematic review should consider the aspects of adverse events relevant to MSC treatment.^19^ Randomized controlled trials (RCTs) would be insufficient to provide evidence of benefits and harms; thus, non-RCT, such as prospective cohort studies with long-term follow up periods should be included.^19^ However, no systematic reviews have investigated adverse events after MSC treatment, even though previous systematic reviews included both RCTs and non-RCTs.^7–9^ Thus, the purpose of this systematic review was (i) to examine the literature on the effects of MSCs in patients with knee OA in the clinical setting and to summarize the current evidence for their potential benefits and harms, and (ii) to examine whether rehabilitation is an effect modifier of effect estimate of MSC treatment. This study would provide a framework for a future high quality study with the aim of developing effective cell-based regenerative rehabilitation in patients with knee OA.

## Results

eFigure 1 shows a flow chart of the study selection. The database search yielded 659 studies, of which 31 met the eligibility criteria. With the citation index, 4 additional studies were found in accordance with the pre-specified inclusion criteria provided in eMethod 1; in total, 35 studies were used in the meta-analysis.

### Study characteristics

Table 1 shows the characteristics of the included studies. Of 35 studies, 21 (60.0%)^20–40^ had a single-arm prospective design, 7 (20.0%)^6,41–46^ had a quasi-experimental design, and the remaining 7 (20.0%)^5,47–52^ were RCTs. From the 35 studies, 2385 patients treated with MSC therapy were included. The mean age across 35 articles was 56.7 ± 6.78 years (36.0–74.5 years). In the 30 studies that reported sex (*n* = 1975 patients), 1119 patients (56.7%) were female. Twenty-nine studies (82.9%)^5,6,20,23–35,37–48,50^ reported the radiographic severity of knee OA (i.e., Kellgren/Lawrence [K/L] grade); however, the eligibility criteria of disease severity differed between studies. The final follow-up period was 3–60 months. Fourteen studies (40.0%)^5,6,20,23–27,33,38,40,42,46,47^ reported funding sources (eTable 1). Of the 35 studies, 25 (73.5%)^5,6,20–27,31–42,45,46,49^ and 2 (5.7%)^47,50^ used autologous and allogeneic MSC intra-articular injection, respectively. The other studies used arthroscopic autologous MSC implantation,^28–30,43,44^ or a combination of these procedures with high tibial osteotomy.^48,51,52^ The rehabilitation program included patients’ education in the pre-MSC treatment phase, gradual increase in weight-bearing using crutches, use of physical therapy modalities, range of motion exercise, and muscle strength exercise (eTable 2). Notably, none of the included studies stratified for the presence of rehabilitation.

**Table 1:** Summary of included studies

Author	Subject population	KL grade	Treatment	Donor	Outcomes	Follow-up	Funding
Single-arm, prospective follow-up studies							
Bui 2014^20^ (Vietnam)	*N* = 21	II–III	SVF injection + PRP	Auto	Lysholm score, VAS pain, MRI	1, 3, 6 M	X
Centeno 2008a^21^ (Unites states)	*N* = 1 (age: 36 y; M)	–	BD-MSC injection (4.56 × 10^7^ cells)	Auto	VAS pain, MRI (cartilage and meniscus volumes)	1, 3 M	–
Centeno 2008b^22^ (Unites states)	*N* = 1 (46 y; M)	–	BD-MSC injection (2.24 × 10^7^ cells)	Auto	VAS pain, functional rating index, ROM, MRI evaluation (cartilage and meniscus volumes)	1, 3, 6 M	–
Davatchi 2011^23^ (Iran)	*N* = 4 (age: 57.8 ± 5.0 y; 50% F)	II–III	BD-MSC injection (8–9 × 10^6^ cells)	Auto	VAS pain, ROM	6 M	X
Davatchi 2016^24^ (Iran)	*N* = 4 (age: 57.8 ± 5.0 y; 50% F)	II–III	BD-MSC injection (8–9 × 10^6^ cells)	Auto	VAS pain, ROM	60 M	X
Emadedin 2012^25^ (Iran)	*N* = 6 (age: 53.8 ± 8.9 y; 100% F)	IV	BD-MSC injection (2.0–2.4 × 10^7^ cells)	Auto	VAS pain, WOMAC, ROM, MRI evaluation	2 W; 1, 2, 6, 12 M	X
Emadedin 2015^26^ (Iran)	*N* = 6 (age: 53.8 ± 8.9 y; 100% F)	IV	BD-MSC injection (2.0–2.4 × 10^7^ cells)	Auto	VAS pain, WOMAC, MRI evaluation	2, 6, 12, 30 M	X
Fodor 2016^27^ (Unites states)	*N* = 6 patients 8 knees (age: 59.0 ± 7.3 y; 83.3% F)	I (*N* = 2) II (*N* = 2) III (*N* = 4)	SVF injection	Auto	VAS pain, WOMAC, ROM, TUG, MRI evaluation	3, 12 M	X
Kim 2015c^28^ (Korea)	*N* = 49 patients, 55 knees (age: 58.1 ± 8.9 y; 52.7% F)	I–II	AD-MSC implantation (4.3 × 10^6^ cells) + AD	Auto	IKDC, Tegner activity scale	26.7 M	–
Kim 2016^29^ (Korea)	*N* = 20 patients, 24 knees (age: 57.9 ± 5.9 y; 45.0% F)	I–II	AD-MSC implantation (4.4 × 10^6^ cells) + AD	Auto	IKDC, Tegner activity scale, MRI evaluation (MOCART and MOAKS)	27.9 M	–
Koh 2013^32^ (Korea)	*N* = 18 (age: 54.6 ± 7.8 y; 66.7% F)	III–IV	AD-MSC injection (1.18 × 10^6^ cells) + PRP	Auto	WOMAC, Lysholm score, VAS pain, MRI evaluation (WORMS)	24.3 M	–
Koh 2014a^30^ (Korea)	*N* = 35 patients, 37 knees (age: 57.4 ± 5.7 y; 60.0% F)	I–II	AD-MSC implantation (3.8 × 10^6^ cells) + AD	Auto	IKDC, Tegner activity scale, arthroscopic evaluation (ICRS grade)	26.5 M	–
Koh 2015^31^ (Korea)	*N* = 30 (age: 70.3 [65–80] y; 83.3% F)	II–III	AD-SVF (4.2 × 10^7^ cells) injection + PRP + AD	Auto	Lysholm, KOOS, VAS pain, K/L grade, arthroscopic evaluation	3, 12, 24 M	–
Michalek 2015^33^ (Czech Republic)	*N* = 1114 (age: 62.0 [19–94] y; 47.8% F)	II–IV	AD-SVF injection (1.6 × 10^6^ cells) + PRP	Auto	Modified KOOS, X-ray, MRI evaluation	17.2 M	X
Orozco 2013^34^ (Spain)	*N* = 12 (age: 49.0 ± 17.3 y; 50.0% F)	II (*N* = 4) III (*N* = 3) IV (*N* = 5)	BD-MSC injection (4.0 × 10^7^ cells)	Auto	VAS pain, Lequesne index, WOMAC, PCI, SF-36	3, 6, 12 M	–
Orozco 2014^35^ (Spain)	*N* = 12 (age: 49.0 ± 17.3 y: 50.0% F)	II–IV	BD-MSC injection (4.0 × 10^7^ cells)	Auto	VAS pain score, Lequesne index, WOMAC, PCI	3, 6, 12, 24 M	–
Pak 2011^36^ (Korea)	*N* = 2 (age: 74.5 ± 6.4 y; 100% F)	–	AD-MSC injection + HA + PRP + CaCl_2_ + dexamethasone	Auto	VAS pain, ROM, MRI evaluation	3 M	–
Sampson 2016^37^ (Unites states)	*N* = 125 (age: 57.0 [23–79] y; 100% F)	III–IV	BMC injection + PRP	Auto	VAS, global patients satisfaction survey	4.8 M	–
Soler Rich 2015^39^ (Spain)	*N* = 50 (age: 57.8 ± 14.1 y; 40.0%F)	II–IV	BD-MSC injectio (4.0 × 10^7^ cells)	Auto	VAS, Lequesne score, WOMAC, MRI evaluation T2 mapping, PCI)	0, 6, 12 M	–
Soler 2016^38^ (Spain)	*N* = 15 (age: 51.1 ± 10.3 y; 60.0% F)	II (*N* = 9) III (*N* = 6)	BD-MSC injection (4.1 × 10^7^ cells)	Auto	VAS, Lequesne score, WOMAC, SF-36, MRI evaluation (T2 mapping)	1 W; 3, 6, 12, 48 M	X
Trajune 2013^40^ (Thailand)	*N* = 5 (age: 57.2 ± 1.92 y; 80.0% F)	II	AAPBSC injection + GFAP concentrate + HA + MCS	Auto	WOMAC, KOOS	1, 6 M	X
Quasi-experimental studies							
Centeno 2014^41^ (Unites states)	I: *N* = 518 (age 54.3 ± 14.1 y) C: *N* = 163 (age 59.9 ± 10.3 y)	I: I (*N* = 223) II (*N* = 145) III/IV (*N* = 102) C: I (*N* = 69) II (*N* = 58) III/IV (*N* = 39)	I: BMC injection + PRP with adipose fat graft C: BMC injection + PRP	Auto	Improvement rating scale, LEFS, NPS	1, 3, 6, 12 M	–
Jo 2014^42^ (Korea)	I-a: Low dose, *N* = 3 (age: 63.0 ± 8.6 y; 66.7% F) I-b: Mid dose, *N* = 3 (age: 65.0 ± 6.6 y; 100% F) I-c: High dose, *N* = 12 (age: 61.0 ± 6.2 y; 83.3% F)	I-a: III (*N* = 2) IV (*N* = 1) I-b: III (*N* = 2) IV (*N* = 1) I-c: III (*N* = 8) IV (*N* = 4)	AD-MSC injection (I-a: 1.0 × 10^7^, I-b: 5.0 × 10^7^, I-c: 1.0 × 10^8^ cells)	Auto	WOMAC, VAS pain, KSS, MRI evaluation (defect size and cartilage volume), arthroscopic evaluation (defect size and ICRS grade), biopsy	1, 2, 3, 6 M	X
Kim 2015a^43^ (Korea)	I: *N* = 17 patients, 17 knees (age: 57.7 ± 5.8 y; 52.9% F) C: *N* = 37 patients, 39 knees (age: 57.5 ± 5.9 y; 62.2% F)	I–II	I: AD-MSC implantation with fibrin glue (3.9 × 10^6^ cells) + AD C: AD-MSC implantation (3.9 × 10^6^ cells) + AD	Auto	IKDC, Tegner activity scale, arthroscopic evaluation (ICRS grade)	28.6 M	–
Kim 2015b^44^ (Korea)	I: *N* = 20 (age: 59.1 ± 3.5 y; 65.0% F) C: *N* = 20 (age: 59.4 ± 3.1 y; 65.0% F)	I–II	I: AD-MSC implantation (4.0 × 10^6^ cells) + AD C: AD-MSC injection (4.0 × 10^6^ cells) + PRP	Auto	IKDC, Tegner activity scale, arthroscopic evaluation (ICRS grade)	28.6 M	–
Koh 2012^45^ (Korea)	I: *N* = 25 (age: 54.2 ± 9.3 y; 68.0% F) C: *N* = 25 (age: 54.4 ± 11.3 y; 68.0% F)	I: 3.3 ± 0.8 C: 2.7 ± 0.7	I: AD-MSC injection (1.89 × 10^6^ cells) + PRP C: PRP	Auto	Lysholm, Tegner activity scale, VAS pain	3, 16.4 M	–
Nguyen 2017^46^ (Vietnam)	I: *N* = 15 (age: 58.6 ± 6.5 y; 80.0% F) C: *N* = 15 (age: 58.2 ± 5.7 y; 80.0% F)	I: II (*N* = 4) III/IV (*N* = 11) C: II (*N* = 5) III/IV (*N* = 10)	I: AD-SVF injection (1.89 × 10^6^ cells) + AM + PRP C: AM + PRP	Auto	WOMAC, modified VAS pain, Lysholm, MRI	1, 6, 12, 18 M	X
Pers 2016^6^ (France)	I-a: Low dose, *N* = 6 (age: 63.2 ± 4.1 y; 50.0% F) I-b: Mid dose, *N* = 6 (age: 65.5 ± 8.1 y; 50.0% F) I-c: High dose, *N* = 6 (age: 65.2 ± 2.3 y; 66.7% F)	I-a: III (*N* = 2) IV (*N* = 41) I-b III (*N* = 1) IV (*N* = 5) I-c III (*N* = 0) IV (*N* = 6)	AD-SVF injection (I-a: 2 × 10^6^, I-b: 10 × 10^6^, I-c: 50 × 10^6^ cells)	Auto	WOMAC, Global knee pain, PGA, KOOS, SAS, SF-36, MRI evaluation	1 W; 3, 6 M	X
Randomized controlled trials							
Gupta 2016^47^ (India)	Cohort 1: I-a (Low dose): *N* = 10 (age: 58.1 ± 8.2 y; 70.0% F) I-b (Mid dose): *N* = 10 (age: 57.3 ± 9.5 y; 80.0% F) C-a: *N* = 10 (age: 54.9 ± 8.3 y; 100.0% F) Cohort 2: I-c (High dose): *N* = 10 (age: 55.0 ± 6.7 y; 80.0% F) I-d (Very high dose): *N* = 10 (age: 54.0 ± 6.7 y; 50.0% F) C-b: *N* = 10 (age: 56.7 ± 5.2 y; 70.0% F)	I-a: II (*N* = 4) III (*N* = 6) I-b: II (*N* = 1) III (*N* = 9) C-a: II (*N* = 3) III (*N* = 7) I-c: II (*N* = 1) III (*N* = 9) I-d: II (*N* = 3) III (*N* = 7) C-b: II (*N* = 2) III (*N* = 8)	I: BD-MSC injection (I-a: 25 × 10^6^, I-b: 50 × 10^6^, I-c: 75 × 10^6^ cells, I-d: 150 × 10^6^ cells) + HA C: HA	Allo	VAS, WOMAC, ICOAP, X-ray, MRI (WORMS)	12 M	X
Koh 2014b^48^ (Korea)	I: *N* = 21 (age: 54.2 ± 2.9 y; 76.2% F) C: *N* = 23 (age: 52.3 ± 4.9 y; 73.9% F)	I: II (*N* = 0) III (*N* = 9) IV (*N* = 12) C: II (*N* = 1) III (*N* = 11) IV (*N* = 11)	I: HTO + AD-MSC implantation + PRP C: HTO + PRP	Auto	Lysholm, KOOS, VAS pain, FTA, arthroscopic evaluation (Kanamiya grade)	24.4 M	–
Lamo-Espinosa 2016^5^ (Spain)	I-a (Low dose): *N* = 10 (age: 65.9 [IQR: 59.5, 70.6] y; 60.0% F) I-b (High dose): *N* = 10 (age: 57.8 [IQR: 55.0, 60.8] y; 20.0% F) C: *N* = 10 (age: 60.3 [IQR: 55.1, 61.1] y; 30.0% F)	I-a: II (*N* = 1) III (*N* = 2) IV (*N* = 7) I-b: II (*N* = 3) III (*N* = 3) IV (*N* = 4) C: II (*N* = 4) III (*N* = 2) IV (*N* = 4)	I: BD-MSC injection (Low dose: 1 × 10^7^ cells; High dose: 1 × 10^8^ cells) + HA C: HA	Auto	VAS, WOMAC, ROM, X-ray, MRI (WORMS)	3, 6, 12 M	X
Varma 2010^49^ (India)	I: *N* = 25 (age: 50.7 ± 5.4 y) C: *N* = 25 (age: 48.2 ± 5.1 y)	–	I: BMC injection + AD C: AD	Auto	VAS pain, OAOS	1, 2, 3, 6 M	–
Vega 2015^50^ (Spain)	I: *N* = 15 (age: 56.6 ± 9.6 y; 60.0% F) C: *N* = 23 (age: 57.3 ± 9.4 y; 66.7% F)	I: II (*N* = 6) III (*N* = 6) IV (*N* = 3) C: II (*N* = 7) III (*N* = 5) IV (*N* = 3)	I: BD-MSC injection (4.0 × 10^7^ cells) C: HA	Allo	VAS pain, WOMAC, Lequesne algofunctional indices, SF-12, MRI evaluation (T2 mapping, PCI)	1 W; 3, 6, 12 M	–
Wakitani 2002^51^ (Japan)	*N* = 24 (I: *N* = 12; C: *N* = 12) (age: 63.0 [49–70] y; 62.5% F)	–	I: HTO + BD-MSC implantation (1.0 × 10^7^ cells) C: HTO + cell free collagen gel-sheet implantation	Auto	Hospital for special surgery knee-rating scale, arthroscopic and histological assessment	16 M	–
Wong 2013^52^ (Singapore)	I: *N* = 28 (age: 53.0 [36–54] y; 54.0% F) C: *N* = 28 (age: 49.0 [24–54] y; 50.0% F)	–	I: HTO + BD-MSC implantation (1.5 × 10^7^ cells) C: HTO	Auto	IKDC, Lysholm, Tegner activity scale, MRI evaluation (MOCART)	6, 12, 24 M	–

*AAPBSC* autologous activated peripheral blood stem cells, *AD* arthroscopic debridement, *AD-MSC* adipose tissue derived mesenchymal stem (stromal) cells, *AD-SVF* adipose tissue derived stromal vascular fraction, *AM* arthroscopic microfracture, *BD-MSC* bone marrow derived mesenchymal stem (stromal) cell, *BMC* bone marrow concentrate, *FTA* femorotibial angle, *GFAP* growth factor addition/preservation, *HA* hyaluronic acid, *HTO* high tibial osteotomy, *ICOAP* intermittent and constant osteoarthritis pain, *ICRS* international cartilage repair society, *IKDC* international knee documentation committee, *IQR* interquartile range, *K/L grade* Kellgren/Lawrence grade, *KOOS* knee osteoarthritis outcome score, *KSS* knee society score, *LEFS* lower extremity functional questionnaire, *MCS* microdrilling mesenchymal cell stimulation, *MOAKS* MRI osteoarthritis knee score, *MOCART* magnetic resonance observation of cartilage repair tissue, *MRI* magnetic resonance image, *NPS* numeric pain scale, *OAOS* osteoarthritis outcome score, *PCI* poor cartilage index, *PGA* patient global assessment, *PRP* platelet-rich plasma, *ROM* range of motion, *SAS* short arthritis assessment scale, *SF-12* short form-12 health survey, *SF-36* short form-36 health survey, *SVF* stromal vascular fraction, *TUG* timed up and go, *VAS* visual analog scale, *WOMAC* Western Ontario and McMaster Universities Osteoarthritis Index, *WORMS* whole-organ magnetic resonance imaging score. *X* indicates presence of funding.

### Risk of bias within studies

A summary of the Downs and Black scale for assessing bias risk is shown in eTable 3. The mean score for all 35 studies was 6.1 ± 2.1 (range, 3–12); 5.5 ± 1.6 for single-arm prospective studies; 6.3 ± 1.0 for quasi-experimental studies; and 7.9 ± 3.2 for RCT. Only two studies^47,50^ received a score of 1, for blinding of participants and assessors who measured key outcomes and concealed randomization of patients. The main differences between RCTs and non-RCTs included the reporting of patients’ recruitment and adequate adjustment for confounders, which is important for assessing the external and internal validities of studies.

### Outcome measures

#### Self-reported knee pain

**Fig. 1:**
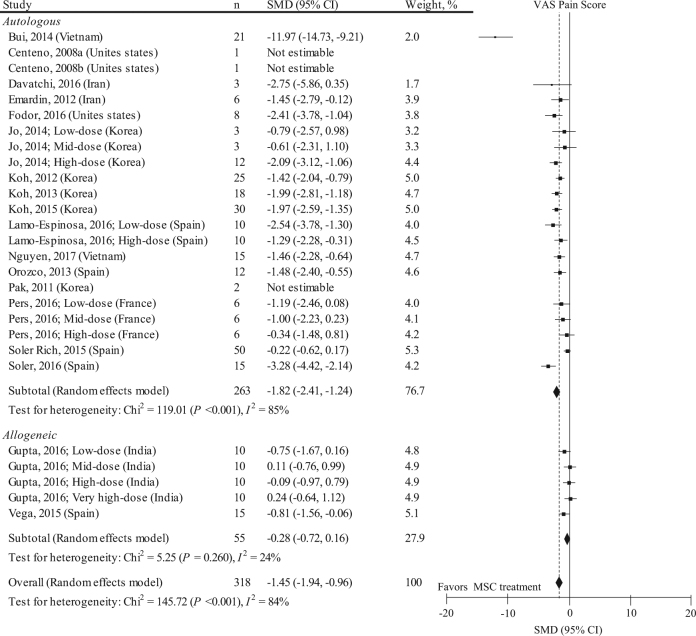
SMD and 95% CI for the VAS pain score between pre and post MSC treatment at final follow-up (*n* = 318). The diamond represents the pooled SMD using the DerSimonian-Laird method. The vertical line at 0 represents no difference. MSC treatment was effective in improving VAS pain score (pooled SMD: −1.45, 95% CI: −1.94, −0.96; *P* < 0.001). SMDs were highly heterogeneous among studies (*I*^2^: 84%; *P* < 0.001)

Nineteen studies with 27 data sets (*n* = 318) reported MSC treatment effects on knee pain by using the visual analog scale (VAS) pain score (Fig. 1). The mean follow-up period in these studies was 14.0 ± 12.9 months. The baseline VAS pain score in these studies was 60.2 ± 13.8 mm. Considering all 19 studies, the pooled standardized mean difference (SMD) on the VAS knee pain was −1.45 (95% confidence interval [CI]: −1.94, −0.96; *P* < 0.001). This statistical value implies a mean difference of 27.6 mm (95% CI: 13.4, 41.9 mm). However, effects estimates were highly heterogeneous among studies (*I*^2^ = 84%). Stratification for donor type (i.e., autologous vs. allogeneic) did not much improve the heterogeneity, but the pooled SMD in autologous MSC was likely to have a larger pain relief effects than those in allogeneic MSC. A meta-regression analysis indicated that a higher score of the Downs and Black scale (i.e., low risk of bias) is significantly associated with a higher (i.e., lower effect) SMD (eTable 4). Among the subitems of the Down and Black scale and SMD, clear patients’ recruitment site was significantly associated with a higher SMD (eTable 5). Rehabilitation (i.e., using physical therapy modalities, range of motion exercise, or muscle strength exercise at least one time) was not an effect modifier of SMD (regression coefficient: 0.451, 95% CI: −1.909, 2.811; *P* = 0.696). Small-study effects were visually observed by two independent reviewers (eFigure 2), and the Egger’s regression test was positive for significant evidence of publication bias (*P* = 0.016). By using the trim-and-fill method, the adjusted SMD was −0.93 (95% CI: −1.29, −0.56; *P* < 0.001).

**Fig. 2:**
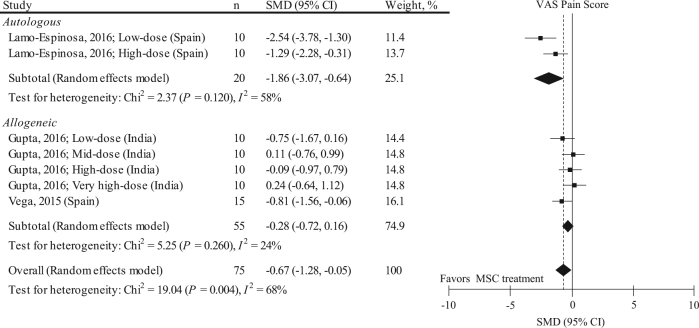
Results of sensitivity analysis representing SMD and 95% CI for the VAS pain score between pre and post MSC treatment at final follow-up in 3 RCTs with 7 data sets (*n* = 75). The diamond represents the pooled SMD using the DerSimonian–Laird method. The vertical line at 0 represents no difference. Including only RCTs attenuates the pain relief effects (pooled SMD: −0.67, 95% CI: −1.28, −0.05; *P* = 0.030) compared to those shown in Fig. 1. SMDs were highly heterogeneous among studies (*I*^2^: 68%; *P* = 0.004)

To address the possibility that effect estimates on VAS pain score and heterogeneity change if only RCTs were included in the meta-analysis, we performed a sensitivity analysis (Fig. 2). Three RCT studies with 7 data sets (*n* = 75) were included, and the follow-up period of all these studies was 12.0 months. The baseline VAS pain score of these studies was 60.4 ± 9.2 mm. Including only RCTs attenuated the pain relief effects (pooled SMD: −0.67, 95% CI: −1.28, −0.05; *P* = 0.030). This statistical value implies a mean difference of 18.1 mm (95% CI: 1.35, 34.8 mm). However, effects estimates were still highly heterogeneous among the studies (*I*^2^ = 68%). Stratification for donor type slightly improved the heterogeneity, and the pooled SMD in autologous MSC was likely to have larger pain relief effects than those in allogeneic MSC. A meta-regression analysis indicated that a higher score in the Downs and Black scale and younger age were significantly associated with higher (i.e., lower effect) SMDs (eTable 6), and blinding of participants and assessors, valid outcome measures, and concealed allocation were significantly associated with higher SMDs (eTable 7). As all the included RCTs did not report a rehabilitation program, the regression coefficient could not be calculated. No small-study effect was visually observed by two independent reviewers (eFigure 3).

#### Self-reported physical function

**Fig. 3:**
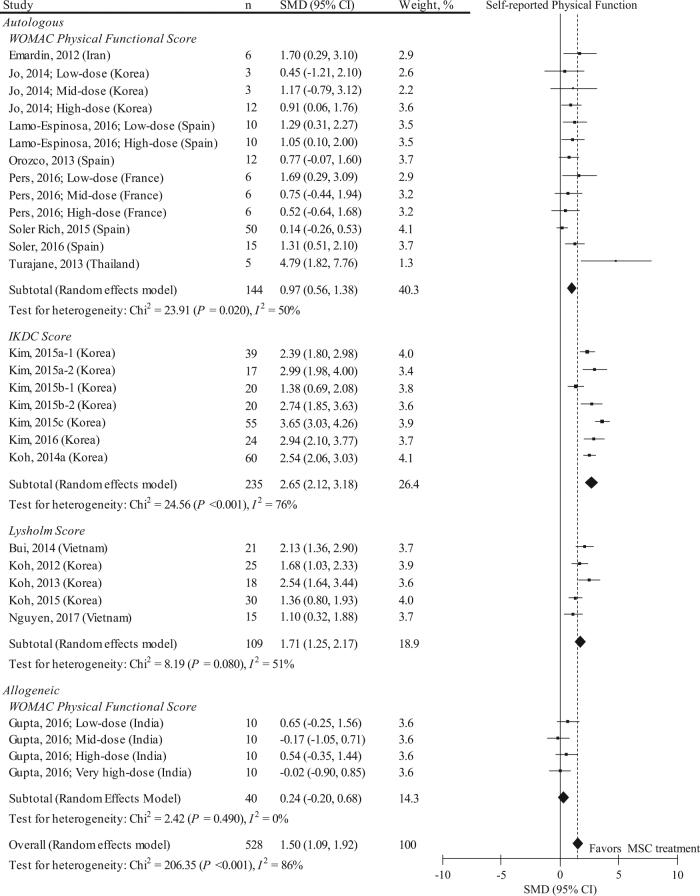
SMD and 95% CI for the self-reported physical functional outcome between pre and post MSC treatment at final follow-up. The diamond represents the pooled SMD using the DerSimonian-Laird method. The vertical line at 0 represents no difference. MSC treatment was effective in improving self-reported physical function (pooled SMD: 1.50, 95% CI: 1.09, 1.92; *P* < 0.001). SMDs were highly heterogeneous among studies (*I*^*2*^: 86%; *P* < 0.001)

Nineteen studies with 29 data sets (*n* = 528) reported MSC treatment effects on self-reported physical function by using the Western Ontario and McMaster Universities Osteoarthritis Index (WOMAC) functional, International Knee Documentation Committee (IKDC), and Lysholm scores (Fig. 3). The mean follow-up period in these studies was 17.0 ± 10.8 months. Considering all 19 studies, the pooled SMD on the self-reported physical function was 1.50 (95% CI: 1.09, 1.92; *P* < 0.001). This statistical value implies a mean difference of 14.7 (95% CI: 9.39, 20.0) in the WOMAC functional outcome (0–100 points); 26.0 (95% CI: 23.1, 28.9) in the IKDC (0–100 points); and 24.1 (95% CI: 19.0, 29.2) in the Lysholm score (0–100 points). However, effects estimates were highly heterogeneous among the studies (*I*^*2*^ = 86%). Pooled SMD in autologous MSC was likely to have a larger functional improvement effects than those in allogeneic MSC. A meta-regression analysis indicated that implantation technique (compared to injection), lower Downs and Black scale score, presence of rehabilitation, and absence of funding source were significant factors associated with higher (i.e., higher effect) SMDs (eTable 8), and blinding of participants, unblinding of assessors, unclear patients’ recruitment site, non-randomization and non-concealed allocation were significant factors associated with higher SMDs. (eTable 9). Notably, performing rehabilitation was a significant effect modifier of SMD (regression coefficient: 0.881, 95% CI: 0.049, 1.712; *P* = 0.039). No small-study effect was visually observed by two independent reviewers (eFigure 4), and the Egger’s regression test was negative for significant evidence of publication bias (*P* = 0.516).

**Fig. 4:**
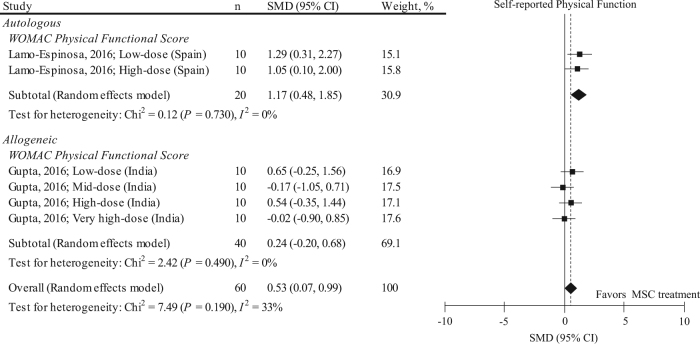
Results of sensitivity analysis representing SMD and 95% CI for the self-reported physical function (WOMAC physical functional score) between pre and post MSC treatment at final follow-up in 2 RCTs with 6 data sets (*n* = 60). The diamond represents the pooled SMD using the DerSimonian-Laird method. The vertical line at 0 represents no difference. Including only RCTs attenuates the effects of MSC in improving WOMAC functional score (pooled SMD: 0.53, 95% CI: 0.07, 0.99; *P* = 0.020) compared to those shown in Fig. 3

As in the VAS pain score, we performed a sensitivity analysis (Fig. 4) and included only RCTs into the meta-analysis for self-reported physical function (*n* = 60). We found that including only RCTs in the meta-analysis attenuated the effects of MSC in improving WOMAC functional score (pooled SMD: 0.53, 95% CI: 0.07, 0.99; *P* = 0.020). The follow-up period in all these studies was 12.0 months. Heterogeneity was much improved because of using a single outcome measure (*I*^2^ = 33%). Stratification for donor type improved the heterogeneity, and pooled SMD in autologous MSC was likely to have a larger functional improvement effects than those in allogeneic MSC. All the included RCTs did not perform rehabilitation. No small-study effect was visually observed by two independent reviewers (eFigure 5).

#### MRI findings in articular cartilage

**Fig. 5:**
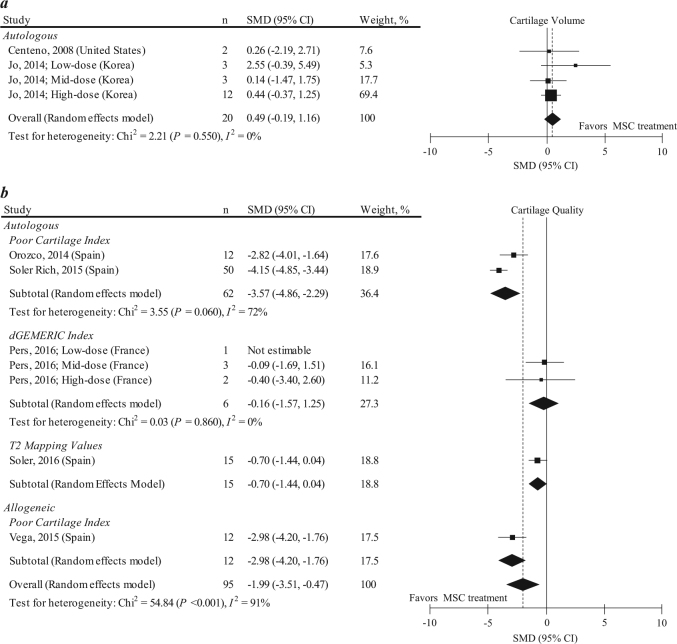
SMD and 95% CI for cartilage volume (**a**) and cartilage quality (**b**) between pre and post MSC treatment at final follow-up. The diamond represents the pooled effect size using the DerSimonian-Laird method. The vertical line at 0 represents no difference. While MSC treatment has a non-significant tendency to improve cartilage volume (pooled SMD: 0.49, 95% CI: −0.19, 1.16; *P* = 0.160), MSC treatment was effective in improving cartilage quality (pooled SMD: −1.99, 95% CI: −3.51, −0.47; *P* < 0.001). SMDs for cartilage quality were highly heterogeneous among studies (*I*^2^: 91%; *P* < 0.001)

Two studies with 4 data sets (*n* = 20) reported the MSC treatment effect on cartilage volume, evaluated using magnetic resonance imaging (MRI; Fig. 5a). The mean follow-up period of these studies was 5.3 ± 1.5 months. In these analyses, two single case reports from the same authors^21,22^ were combined, as these case reports included patients with a similar clinical status. The pooled SMD on the cartilage volume was 0.49 (95% CI: −0.19, 1.16; *P* = 0.160), a non-significant small effect size. Excluding the combined two case reports resulted in similar results (pooled SMD: 0.51, 95% CI: −0.23, 1.26; *P* = 0.180).

The 5 other studies with 7 data sets (*n* = 95) reported MSC treatment effects on cartilage quality by using the poor cartilage index (PCI), dGEMERIC index, and T2 mapping values, evaluated using MRI (Fig. 5b). The mean follow-up period in these studies was 16.3 ± 15.4 months. The pooled SMD on the cartilage quality was −1.99 (95% CI: −3.51, −0.47; *P* < 0.001), a significantly heightened effect size (SMD ≥ 0.8), with high heterogeneity (*I*^2^ = 91%). When the pooled SMD was evaluated in each outcome measure, it became higher in the PCI but became insignificant in the dGEMERIC index, and heterogeneity improved markedly. A meta-regression analysis indicated that the presence of funding source was a significant factor associated with a higher (i.e., lower effect) SMD (eTable 10). No small-study effect was visually observed from funnel plots by two independent reviewers (eFigures 6 and 7).

### Adverse events

Of 35 studies, 17 (48.6%) reported adverse events related to MSC treatment. Adverse events included knee pain or swelling. eFigure 8 summarizes the event rates with their 95% CIs. Owing to the large clinical and statistical heterogeneity among the studies, we did not pool the adverse event rates. In 10 studies that reported timing of adverse event,^5,6,31,32,34,37–39,45,50^ knee pain or swelling occurred within 1 week after MSC treatment; these symptoms were treatable with pain medication.

### Summary of quality of evidence

Table 2 shows a summary of evidence according to the GRADE approach.^12^ The effects estimate was downgraded in all outcome measures. None of these effects estimates were upgraded. Each meta-analysis scored 1 (very low) or 2 (low) with the GRADE approach, indicating very little (i.e., the true effect is likely to be substantially different from the effect estimate) or limited (i.e., the true effect may be substantially different from the effect estimate) confidences of the effects estimate.^12^

**Table 2:** Summary of body of evidence according to the GRADE’s approach

Outcome	SMD (95% CI)	Study design	Sample size	Downs and black scale	Heterogeneity	Effect of rehab.	Level of evidence (GRADE)
VAS pain score	−1.45 (−1.94, −0.96)	12 × Within-subject repeated design 8 × Quasi-experimental design 7 × RCT	*n* = 318	7.2 ± 2.6 (7 [4–12]) points	*I*^2^ = 84%	Unclear	⊕ ⊖ ⊖ ⊖ Very low^a,b,d^
VAS pain score (Trim-and-fill)	−0.93 (−1.29, −0.56)						⊕ ⊖ ⊖ ⊖ Very low^a,b^
VAS pain score (sensitivity analysis)	−0.67 (−1.28, −0.05)	7 × RCT	*n* = 75	10.9 ± 2.0 (12 [8–12]) points	*I*^2^ = 68%	Unclear	⊕ ⊖ ⊖ ⊖ Very low^b,c,d^
Self-reported physical function	1.50 (1.09, 1.92)	11 × Within-subject repeated design 12 × Quasi-experimental design 6 × RCT	*n* = 528	7.2 ± 2.0 (7 [4–12]) points	*I*^2^ = 86%	Significant effect modifier^e^	⊕ ⊖ ⊖ ⊖ Very low^a,b^
Self-reported physical function (sensitivity analysis)	0.53 (0.07, 0.99)	6 × RCT	*n* = 60	10.7 ± 2.1 (12 [8–12]) points	*I*^2^ = 33%	Unclear	⊕ ⊕ ⊖ ⊖ Low^c,d^
Cartilage volume	0.49 (−0.19, 1.16)	1 × Within-subject repeated design 3 × Quasi-experimental design	*n* = 20	6.3 ± 1.5 (7 [4–7]) points	*I*^2^ = 0%	Unclear	⊕ ⊖ ⊖ ⊖ Very low^a,c,d^
Cartilage quality	−1.99 (−3.51, −0.47)	3 × Within-subject repeated design 3 × Quasi-experimental design 1 × RCT	*n* = 95	7.4 ± 2.1 (7 [5–12]) points	*I*^2^ = 91%	Unclear	⊕ ⊖ ⊖ ⊖ Very low^a,b,c,d^

^a^Downgraded for risk of bias (most of included studies scored less than 8 points on the Downs and Black scale)

^b^Downgraded for inconsistency (results were highly heterogeneous across included studies)

^c^Downgraded for imprecision (clinical action would differ if true SMD is the upper or the lower boundary of the 95% CI)

^d^Downgraded for publication bias (Egger’s regression test was positive or unable to determine because of a few included studies [<10 data set])

^e^Presence of rehabilitation (physical therapy modalities, range of motion exercise, or muscle strength exercise) is a significant effect modifier on the SMD for self-reported physical function (regression coefficient: 0.881, 95% CI: 0.049, 1.712

*P* = 0.039; see eTable 8 in the Supplementary Materials)

## Discussion

This systematic review and meta-analysis found that MSC treatment significantly improved knee pain and self-reported physical function in patients with knee OA. While MSC treatment has an insignificant tendency to improve cartilage volume, MSC treatment significantly improved cartilage quality. However, these data should be interpreted with caution because the quality of evidence was “very low” to “low” according to the GRADE approach because of the poor study design, high risk of bias, large heterogeneity, and wide 95% CI of the pooled SMD. Sensitivity analyses showed that these GRADE ratings were comparable even if we only included RCTs in the meta-analysis; therefore, the true effect is likely to be substantially different from the effects estimate.^12^ Detail information about rehabilitation was lacking, but rehabilitation was a significant effect modifier of MSC treatment on self-reported physical function. We suggest that more high quality RCTs with stratification for rehabilitation are needed to facilitate a foundation of effective MSC therapy and regenerative rehabilitation.

The search strategies used in this study provide a more comprehensive assessment of relevant articles by adding new findings to the recent meta-analysis for the clinical efficacy of MSCs transplantation for knee OA and focal cartilage defect up to a maximum 24 months follow-up.^10^ Indeed, the current meta-analysis further added 28 non-RCTs and 4 RCTs to the previous meta-analysis,^10^ which enable us to examine the latest evidence of both benefits and harms of MSCs treatment on degenerative knee OA with a longer follow-up period that cannot be adequately determined by reviewing only RCTs.^19^

We found that the pooled effect size on the VAS pain score exceeded the effects of nonsteroidal anti-inflammatory drugs and corticosteroid injections,^53,54^ consistent with previous meta-analyses.^7,9,10^ The mean differences after intervention were ≥10% for both pain and self-reported physical function,^55^ exceeding the minimum for clinically important differences, and meeting the responder criteria of the Outcome Measures in Rheumatology Clinical Trials and Osteoarthritis Research Society International. However, we found a large heterogeneity among studies, which was partly explained by the level of risk of bias, cell donor type, and study design. Including only RCTs, which has a lower risk of bias than non-RCTs, in the meta-analysis attenuated the effects of MSC treatment in improving knee pain and self-reported physical function, supporting this interpretation. The observed effects from RCTs had a wide 95% CI, and clinical action would differ if the true SMD was the upper or lower boundary of the 95% CI. This suggests the need for a larger number of RCTs to elucidate whether MSC treatment can provide clinical benefit to patients with knee OA.

The strength of this meta-analysis is that we estimated pooled SMD for structural outcomes of articular cartilage evaluated by MRI. This effect estimate was based on only 2 non-RCTs with 4 data sets, raising the need for high quality RCTs for examination of the structural modifying effects of MSC treatment. We found a discrepancy between MSC efficacy on cartilage quality and MSC efficacy on cartilage quantity (volume). While MSC treatment improved cartilage quality, it did not significantly improve cartilage volume. Although these results should be interpreted cautiously because the studies that evaluated cartilage quality differed from that evaluated cartilage volume, we found that MSC treatment may have a limited therapeutic effect on cartilage volume. Three of these 4 data sets were based on data from patients with severe knee OA (K/L grade ≥3), which may cause limited efficacy in improving cartilage volume. Furthermore, the mean follow-up period in these studies was within 6 months, which might be too short to show a biological effect. One high quality study^42^ found that MSC injection particularly improved knee pain when a relatively large number of MSCs was used, but a significant increase in cartilage volume did not accompany this pain reduction, indicating that improved knee pain is not necessarily attributable to increased cartilage volume. Although this meta-analysis only included outcome measures for articular cartilage, some included studies found that MSC treatment improved subchondral bone edema^25,26,46^ and meniscus thickness,^36^ which are predictors of knee pain severity.^56^ Improved knee pain after autologous chondrocyte implantation on cartilage defects moderately correlated with bone edema, but not the cartilage structure evaluated using MRI.^17^ Further studies that investigate the mechanism of pain reduction after MSC treatment in patients with knee OA would be of interest.

Physical factors regulate MSC differentiation and tissue development, pointing to a potential therapeutic strategy for enhancing the MSCs injected into the knee joint.^13,14^ Weight-bearing might influence the structural outcome evaluated by MRI in the postoperative phase of autologous chondrocyte implantation.^17,18^ The mean follow-up period after MSC treatment was 3–60 months in the included studies, which includes some rehabilitation and physical activity programs in the post-MSC treatment phase. These post-MSC rehabilitations might affect the effects of cell-based therapy. Indeed, the presence of rehabilitation was a significant effect modifier of SMD on self-reported physical function. Although the presence of a rehabilitation program was not a significant effect modifier of the estimated effect on VAS pain score, rehabilitation does not necessarily have no impact; the lack of statistical power due to a small number of studies in the meta-analysis^19^ and the lack of details of rehabilitation program in each article may explain this absence. As physiological stimulation such as moderate level exercise,^57^ ultrasound irradiation,^58^ and mechanical loading after joint distraction^59^ may enhance cartilage regeneration after MSC injection in a preclinical study, applying exogenous stimulation may be one strategy for enhancing the injected MSCs. This point is particularly important because the lower boundary of the 95% CI of SMD on knee pain and physical function corresponds to the lower effect size in the meta-analysis of RCTs. As all the included RCTs did not report (perform) rehabilitation and none of the included non-RCTs stratified for rehabilitation program, investigating the effects of rehabilitation on the SMD of MSC treatment would be of interest in future studies. Rehabilitation programs was differed among the included studies; thus, this review highlights the need for a standardized rehabilitation program that encompasses at least weight-bearing schedule, range of motion exercise, and muscle strength exercise, which would influence the therapeutic effect of MSCs to facilitate further comparisons among studies. The implementation of longitudinal activity-based questionnaires might help address this question.

We observed a large heterogeneity of adverse event rates among the included studies; this observation limits our ability to summarize the adverse event rate. The causes of heterogeneity in this study are unclear. Detailed reports on adverse events are sparse, which may have contributed to the heterogeneity. Nevertheless, we found only minor adverse events (knee pain/swelling) after MSC treatment, indicating that benefits may outweigh harms of MSC treatment of knee OA. These findings can be achieved by reviewing the data from both non-RCTs and RCTs, which is the strength of the present meta-analysis. Most adverse events occurred within 1 week following MSC treatment. Conversely, pain or swelling that persists for more than 1 week should be interpreted as a rare and potentially severe adverse event that might contribute to arthrogenic muscle inhibition.^60^ Close attention to adverse events may be key to the clinical success in optimizing post-MSC treatment of knee OA.

Autologous MSCs are a widely selected source to minimize the immune response and an excellent therapeutic option for treating OA. Most included trials used autologous MSCs to eliminate immune rejection, while 2 of 35 articles attempted to investigate the potential application of allogeneic MSCs.^47,50^ No observed severe adverse event indicates the safety of allogeneic MSCs for applying knee OA. The present meta-analysis revealed that the therapeutic effects of VAS pain score and self-reported physical function were likely higher in autologous than in allogeneic MSCs. However, direct comparisons of the therapeutic effects between autologous and allogeneic MSCs are difficult because these are based on data from different studies. Moreover, two of the studies of allogeneic MSCs were RCTs, which had lower risks of bias than those of autologous MSCs, which might have contributed to the lower therapeutic effect. Thus, direct comparison between autologous and allogeneic MSCs in the same trial would be of interest.

This systematic review included patients with knee OA diagnosed either radiographically or clinically, and excluded those with a focal cartilage defect. Thus, the observed effect of MSCs on clinical outcomes may not hold true in patients with focal cartilage defects. As knees with OA have diffuse cartilage loss rather than an isolated cartilage lesion, several researchers have sought to assess the effect of inter-articular MSC injections rather than implantation to a focal lesion. Whereas MSC implantation on focal cartilage defects in both preclinical and clinical studies is effective in cartilage repair, the cartilage repair effects of intra-articular injection is controversial.^61^ We found that the type of treatment was a strong effect modifier of MSC treatment on physical function. It should be highlighted that 2 studies failed to detect a clear dose-response relationship between injected MSC and cartilage volume^42^ and cartilage quality;^6^ thereby no effects estimates were upgraded in the GRADE approach. Mamidi *et al*. recently suggested that investigating post-transplanted MSC behavior and how to enhance the potency of the transplanted MSCs are the major challenges to be directly solved in future research.^4^ We could not address post-injected MSC behavior in the diseased microenvironment; investigating the kinematics of injected MSCs is needed to enhance their disease-modifying effects.

The present study has some limitations. First, this meta-analysis included non-RCTs with 3 case reports. As non-RCTs would have greater bias and more confounders than RCTs, evaluating MSC efficacy using only RCTs might be preferable.^19^ Thus, we performed a sensitivity analysis and calculated the effect estimate based on RCTs. Meta-analyses that include non-RCTs can provide evidence of effects that are difficult to detect using a RCT, such as long-term effects and adverse events. Evaluating the beneficial and harmful effects of MSC treatment would be needed to make decisions about the clinical utility of MSC treatment. As discussed previously, as no RCTs have performed rehabilitation, the present meta-analysis, which included non-RCTs, could shed light on the importance of rehabilitation as a new strategy for enhancing functional improvement after MSC treatment and would set a basis for future high quality RCTs. Second, this meta-analysis included 35 studies, but few studies were available for use in the meta-analysis of structural outcomes. This dearth is attributable to the absence of a standard system for evaluating cartilage regeneration. Many studies that use MRI to evaluate cartilage regeneration are only qualitative;^20,25–27,33,36^ using validated imaging outcomes would be integral for scientifically validating cell-based therapies and precipitously advancing efficacy.^62^ Third, the pooled SMD included the effects of cointervention such as PRP with injected or implanted MSC. PRP improves knee pain and physical function in patients with knee OA,^63^ and has a similar effect to MSC injection;^45^ the pooled SMD might be attributed to the cointervention. Nevertheless, we confirmed that use of PRP was not a significant predictor of the pooled SMD (data not shown). Fourth, many studies included in this meta-analysis were performed by the same group of investigators.^28–32,43–45,48^ Thus, caution is required when interpreting the effect estimate, and further studies from different investigators are needed to elucidate the effects of MSCs on knee OA. Finally, a protocol for this systematic review has not been registered. However, protocol registration was not associated with outcome reporting bias in the meta-analysis,^64^ and the outcome measures were extracted according to the highest rank on the pain and functional outcome hierarchy, determined a priori*.*^65,66^

In conclusion, MSC treatment improves knee pain, physical function, and cartilage quality, without any severe adverse events. However, evidence for these outcomes that are considered critical for clinical decision making was “very low” to “low” according to the GRADE system because of the poor study design, high risk of bias, large heterogeneity, and wide 95% CI of the effects estimate. These GRADE ratings were similar even if only high quality RCTs were included in the meta-analysis. Detail information about rehabilitation is lacking; therefore, the role of rehabilitation in MSC treatment in patients with knee OA is unclear. However, rehabilitation was a significant effect modifier of better MSC treatment on self-reported physical function, supporting a concept of the newly born field, regenerative rehabilitation. Integration of rehabilitation into MSC-based therapy may be beneficial at least in improving physical function. These findings would help researchers and clinicians in designing future high quality clinical trials.

## Methods

This study was conducted in accordance with the Preferred Reporting Items for Systematic reviews and Meta-Analyses (PRISMA) statement,^67^ PRISMA protocols (PRISMA-P),^68^ meta-analysis of observational studies in epidemiology (MOOSE) checklist,^69^ and Cochrane handbook for systematic reviews of interventions.^19^ A detailed protocol for this systematic review has not been previously published and registered.

### Literature search and study selection

The electronic databases of PubMed, Physiotherapy Evidence Database (PEDro), Cumulative Index to Nursing and Allied Health Literature (CINAHL), and Cochrane Central Register of Controlled Trials were used. Searches used combined key terms, including “osteoarthritis, knee,” “transplantation,” “stem cells,” and “stromal cells,” using Medical Subject Headings terms. A database search strategy and determining inclusion are provided in the eMethods 1 and 2.

### Outcome measures and data extraction

The primary outcomes in this review were (i) pain, (ii) self-reported physical function, (iii) structural outcomes of articular cartilage evaluated using MRI, and (iv) adverse events relevant to MSC treatment. Two reviewers independently extracted the data regarding authors, country, study design (single-arm, prospective follow-up studies, quasi-experimental studies, and RCTs), subject population, K/L grade, treatment, cell donor type, outcome measures, follow-up period, rehabilitation program, and funding sources using standardized data forms. When an article reported outcomes using multiple pain and functional scales, we used only the scale with the highest rank on the pain and functional outcome hierarchy, in accordance with previous recommendations^65,66^ and meta-analyses^70^ (eMethod 3).

### Data analysis

Percent agreement of duplicate study removal and interrater reliability of title/abstract and full-text screening between the two reviewers were evaluated. For the meta-analysis, pooled estimates and 95% CIs for SMDs for changes in outcomes were calculated using the DerSimonian-Laird method.^71^ The SMD was calculated for paired samples using the within-patient change for patients treated with MSC divided by the pooled standard deviation (SD). Formulae for calculating the pooled SD and pooled SMD are shown in eMethod 5. The meta-analyses were performed using Review Manager Version 5.3 (Nordic Cochrane Center, Cochrane Collaboration, Copenhagen, Denmark). We used a forest plot to represent the meta-analysis results in accordance with a previous study.^72^ The size of the SMD was interpreted using Cohen’s d^73^ (<0.5: small effect size, 0.5–0.8: moderate effect size, and ≥0.8: large effect size). As a clinical frame of reference, a small effect is equivalent to the effect of non-steroidal anti-inflammatory drugs on knee pain in OA trials.^53^ A moderate effect is equivalent to the effect of corticosteroid injections on knee pain.^54^ When mean and SD values were not directly reported in an article, they were calculated from other available data, if possible (eMethod 6). To test for publication bias, we used a funnel plot and Egger’s test,^74^ where publication bias is the tendency for positive trials to be published and the tendency for negative or null trials to not be published. We interpreted *P*-values of <0.10 to indicate the existence of publication bias, as practiced by a previous study.^74^ When studies are relatively few, the power of the test is too low to distinguish chance from real asymmetry; we tested for publication bias only when least 10 studies were included in the meta-analysis,^19^ and if present, adjustment was planned using a trim-and-fill method.^75^ As SMD would be difficult to interpret in a clinical context, the mean differences in pain and functional outcomes were also calculated and compared with minimum clinically important difference (eMethod 7). Furthermore, we performed prespecified sensitivity analyses to provide pooled SMD with 95% CI by using the data from RCTs only.

Study heterogeneity was assessed using the *I*^2^ statistic and Q statistic.^76^ If *I*^2^ was ≥50, random effects meta-regression was performed using the certain parameters selected a priori including the presence of rehabilitation, defined when patients were treated using physical therapy modalities, range of motion exercise, or muscle strength exercise at least one time after MSC treatment (eMethod 8). Adverse events were evaluated in each study, and adverse event rates were calculated from the numbers of events and sample sizes by using the Comprehensive Meta-Analysis software (Biostat, Inc., Englewood, NJ, USA). All other statistical analyses were performed using JMP Pro 12.2 (SAS Institute, Cary, NC, USA).

### Additional methods

Additional methods for assessment of risk of bias and GRADE approach are provided in eMethods in the Supplement.

### Data availability

Data available on request from the authors.

## Electronic supplementary material

Supplementary Materials

## Electronic supplementary material

**Supplementary information** accompanies the paper on the *npj Regenerative Medicine* website (10.1038/s41536-018-0041-8).
