# Supplementary material for: Effectiveness of mesenchymal stem cells for treating patients with knee osteoarthritis: a meta-analysis toward the establishment of effective regenerative rehabilitation
Source: NPJ Regen Med. 2018 Sep 17;3:15. doi: 10.1038/s41536-018-0041-8 (PMC6141619; doi:10.1038/s41536-018-0041-8)
Supplement: Supplementary file 1 — Supplementary Materials [file 41536_2018_41_MOESM1_ESM.docx]

**Supplementary Online Content**

eMethod 1: Literature search keywords 2

eMethod 2: Determining inclusion 3

eMethod 3: Hierarchy of outcome measures and data extraction　　　　　　　　　　　　　 　　　　　4

eMethod 4: Assessment of risk of bias　　　　　　 　　　　　　　　　　　　　　　　　　　　　　5

eMethod 5: Calculation of pooled SMD using a DerSimonian-Laird method　　　　　　　　　　　　　　6

eMethod 6: Imputation methods for missing data of standard deviation　　　　　　　　　　　　　 7

eMethod 7: Minimum clinically important differences in pain and functional outcome measures　　　 　8

eMethod 8: Meta-regression analysis　　　　　 　　　　　　　　　　　　　　　　　　 　　　　9

eMethod 9: Quality assessment of evidence by using the GRADE approach　　　　　　　　　　　　　 10

eTable 1. Funding source　　　　 　　　　　　　　　　 　　　　　 　　　　11–12

eTable 2. Summary of rehabilitation programs　　　　 　　　　　　　　　　　　　　　 　　　　13–18

eTable 3. Downs and Black scale score in each study　　 　　　　　　　　　　　 19–20

eTable 4. Meta-regression analysis of study characteristics on

the effects estimate for the VAS pain score　　 21

eTable 5. Meta-regression analysis estimating the impact of each risks of bias

in the Downs and Black scale on the effects estimate for the VAS pain score　　　　　 22

eTable 6. Meta-regression analysis of study characteristics on

the effects estimate for the VAS pain score in 3 RCTs with 7 data sets　　　　 23

eTable 7. Meta-regression analysis estimating the impact of each risks of bias in the Downs and Black

scale on the effects estimate for the VAS pain score in 3 RCTs with 7 data sets　　 24

eTable 8. Meta-regression analysis of study characteristics on the effects estimate

for the self-reported functional score　　　　　　　　　　　 　　25

eTable 9. Meta-regression analysis estimating the impact of each risks of bias in

the Downs and Black scale on the effects estimate for the self-reported functional measures　　 26

eTable 10. Meta-regression analysis of study characteristics on the effects estimate for the cartilage quality 27

eFigure 1. Review flow diagram　　　　　　　　　　 28

eFigure 2. Funnel plot representing publication bias across 19 studies with 27 data sets

investigating MSC treatment on VAS knee pain score　　　　　　　　　　 29

eFigure 3. Funnel plot representing publication bias across 3 RCTs with 7 data sets

shows a comparison of the effects of MSC treatment on VAS knee pain score 30

eFigure 4. Funnel plot representing publication bias across 19 studies with 29 data sets

investigating MSC treatment on self-reported physical function.　　　　　　　　　　 31

eFigure 5. Funnel plot representing publication bias across 2 RCTs with 6 data sets

shows a comparison of the effects of MSC treatment on self-reported physical function　　 32

eFigure 6. Funnel plot representing publication bias across 4 studies with 4 data sets

investigating MSC treatment on cartilage volume　 　　　　　　　　　 33

eFigure 7. Funnel plot representing publication bias across 5 studies with 7 data sets

investigating MSC treatment on cartilage quality　　　　　　　　　　 34

eFigure 8. Adverse event rate and 95%CI in each included study　　　　　　　　　　 35

Supplementary References　　　　　　　　　　　　　　　　　　　　　　　　　　　　 36–38

This supplementary material has been provided by the authors to give readers additional information regarding their work.

**eMethod 1: Literature search strategy**

Search keywords comprised of synonyms of *knee osteoarthritis* and *stem cells*. The following search strategy was used in each database.

PubMed

#1 osteoarthritis, knee [MeSH] AND transplantation [MeSH]

#2 osteoarthritis, knee [MeSH] AND stem cells [MeSH]

#3 osteoarthritis, knee [MeSH] AND stromal cells [MeSH]

PEDro

#1 osteoarthritis, knee, transplantation

#2 osteoarthritis, knee, stem cells

#3 osteoarthritis, knee, stromal cells

CINAHL

#1 osteoarthritis, knee AND transplantation

#2 osteoarthritis, knee AND stem cells

#3 osteoarthritis, knee AND stromal cells

Cochrane Library (CENTRAL)

#1 osteoarthritis, knee, transplantation

#2 osteoarthritis, knee, stem cells

#3 osteoarthritis, knee, stromal cells

Google Scholar was also used as a complementary search engine. In addition, a manual search of the reference lists of past systematic reviews was performed. Furthermore, citation searching was performed on the original record by using the Web of Science. These citation indexes are recommended by the Cochrane Handbook[^1^](#_ENREF_1).

Studies that met the following criteria were included: (i) published in a peer review journa; (ii) written in English; (iii) had a RCT, controlled clinical trials, crossover studies, case control studies, and case series studies; (iv) patients were diagnosed as having OA in the tibiofemoral joint; and (iv) MSC administered to at least one treatment group. Knee OA needed to be defined either radiographically or clinically by using some established existing criteria for OA such as the American College of Rheumatology criteria[^2^](#_ENREF_2). Including non-RCTs enables to evaluate adverse event that cannot be evaluated using only RCTs. No restrictions were set on study dates, severity of knee OA, or follow-up duration. For each electronic database, the endpoint was August 2017.

**eMethod 2: Determining inclusion**

Two reviewers (HI and TI) independently assessed eligibility in accordance with the Cochran Handbook[^1^](#_ENREF_1). HI and TI correspond to content expert and methodologist, respectively. The reviewers screened the title and abstracts yielded by the search. Full manuscripts of the articles that met the eligibility criteria were then obtained and reviewed. During these processes, the reviewer prepared and used simple predesigned Google spreadsheets to assess eligibility by extracting study features.

**eMethod 3: Hierarchy of outcome measures and data extraction**

Hierarchy of pain outcome measures

1. Global knee pain score (visual analog or Likert scales)

2. Knee pain on walking (visual analog or Likert scales)

3. WOMAC pain score

4. Knee pain during activities other than walking (visual analog or Likert scales)

Hierarchy of physical functional outcome measures

1. WOMAC physical functional score (visual analog or Likert scales)

2. IKDC

3. Lysholm knee scoring scale

4. KOOS physical functional score

Although no standard structural outcomes have been established yet, we used cartilage volume as quantitative measurement and T2-basis outcomes as qualitative measurements evaluated using MRI according to expert recommendation in the OARSI[^3^](#_ENREF_3)^,^[^4^](#_ENREF_4). Cartilage quality would be complementary when evaluating cartilage structure, because it helps distinguish pathology from normal reparative process such as cartilage swelling particularly in mild knee OA[^5^](#_ENREF_5). When multiple time points were reported either in a particular report of a study or over the course of several articles from the same study, the longest follow-up period on MSC treatment was considered. By using the longest follow-up period, means and standard deviations of primary outcomes before and after MSC treatment were extracted when available. The same reviewers (HI and TI) also extracted the following information to Google spreadsheets from each study: authors, country, study design (single-arm, prospective follow-up studies, quasi-experimental studies, and RCTs), subject population, K/L grade, treatment, cell donor type, outcome measures, follow-up period, rehabilitation program, and funding sources. Disagreements between two reviewers were discussed and consensus was archived. Authors were contacted when data were not reported or unclear.

**eMethod 4: Assessment of risk of bias**

Two reviewers (HI and TI) independently evaluated the risk of bias of each study by using the Downs and Black scale[^6^](#_ENREF_6) that is slightly modified to include only 13 items (bias: 7 items; confounding: 6 items) to assess internal validity (minimum: 0 point, maximum: 13 points). All items were scored 1 for fulfilling the criterion or 0 if the criterion was not filled. Publications that did not provide sufficient details to fulfill the criterion were also given a 0 for being undetermined in accordance with the original index of the Downs and Black scale.[^6^](#_ENREF_6) Rewarding a predefined criterion with 1 point suggests that this criterion has a low risk of bias. This scale is a useful tool for assessing risk of bias in observational studies[^7^](#_ENREF_7) and the methodological quality of both RCT and non-RCT of treatment.[^1^](#_ENREF_1) After discussions to resolve disagreements, a consensus score was arrived in each study.

**eMethod 5: Calculation of pooled SMD using a DerSimonian-Laird method**

We calculated a pooled SMD by using a DerSimonian-Laird method according to the previous study[^8^](#_ENREF_8). First, the pooled standard deviation in each study was calculated by using the following formula.

 　　　　　 $N_{i}=n_{1i}+n_{2i}$ (1)

$S_{i}=\sqrt{\frac{(n_{1i}-1)sd_{1i}^{2}+(n_{2i}-1)sd_{2i}^{2}}{N_{i}-2}}$ (2)

*N_i_* = Pooled sample size; n_1_*_i_*: Sample size in the experimental group (post-MSC treatment); n_2_*_i_*: Sample size in the control group (pre-MSC treatment); *S_i_*: Pooled standard deviation; sd_1_*_i_*: Standard deviation in the experimental group; sd_2_*_i_*: Standard deviation in the control group.

Subsequently, the SMD in each study was calculated using Hedges’ adjusted g which is similar to Cohen’s d but includes an adjustment for small sample bias.

$SMD_{i}=\frac{m_{1i}-m_{2i}}{S_{i}}\left(1-\frac{3}{4n_{i}-9}\right)$ (3)

$SE\left\{SMD_{i}\right\}=\sqrt{\frac{N_{i}}{n_{1i}n_{2i}}+\frac{SMD_{i}^{2}}{2\left(N_{i}-3.94\right)}}$ (4)

*N_i_* = Pooled sample size; n_1_*_i_*: Sample size in the experimental group (post-MSC treatment); n_2_*_i_*: Sample size in the control group (pre-MSC treatment); *m_1i_*: Mean value in the experimental group; *m_2i_*: Mean value in the control group; *S_i_*: Pooled standard deviation; SE (*SMD_i_*): Standard error of *SMD_i_*; *SMD_i_*: Pooled standard mean difference.

Using individual SMD, pooled SMD was calculated using a DerSimonian-Laird method with adjustment for weight in each study.

$SMD_{DL}=\frac{\sum\left(weight_{i}\times SMD_{i}\right)}{\sum weight_{i}}$ (5)

$Weight_{i}=\frac{1}{SE\left\{SMD_{i}\right\}^{2}+\tau^{2}}$ (6)

SE (*SMD_i_*): Standard error of *SMD_i_*; *SMD_i_*: Pooled standard mean difference. *SMD_DL_*: Pooled standard mean difference calculated using a DerSimonian-Laird method.

**eMethod 6: Imputation methods for missing data of standard deviation**

When mean and SD values were not directly reported in the article, they were calculated from other available data when possible; for example, the standard error was converted to the SD, or mean and SD values were estimated from the figure. For the significant findings reported with a non-exact *P*-value (i.e. *P* < 0.05 or < 0.01), a conservative approach was taken and we assigned *P*-values of 0.05 or 0.01[^1^](#_ENREF_1).

When individual SD data were missed and interquartile ranges were reported, we calculated the SD by using the following formula[^9^](#_ENREF_9). The width of the interquartile of a standard normal distribution is 1.35 SD; therefore, dividing by 1.35 estimates the SD.

$SD=\frac{IQ_{upper}-IQ_{lower}}{1.35}$ (7)

SD: Standard deviation; IQ: Interquartile range.

**eMethod 7: Minimum clinically important differences in pain and functional outcome measures**

We calculated the mean difference as follows:

$MD_{i}=m_{1i}-m_{2i}$ (8)

*m_1i_*: Mean value in the experimental group; *m_2i_*: Mean value in the control group; *MD_i_*: Pooled mean difference.

Where possible, the pooled mean differences were compared with the minimum clinically important difference in each outcome measure shown as follows:

1. Global knee pain score (visual analog scale): 19.9 mm after non-steroidal anti-inflammatory drug (NSAID)[^10^](#_ENREF_10).
2. WOMAC pain score (Likert scale): 22.9–36.0 points after total knee arthroplasty^[11](#_ENREF_11)^.
3. WOMAC physical functional score (Likert scale): 9.1 points after NSAID[^10^](#_ENREF_10) and 14.4–21.4 points after total knee arthroplasty^[11](#_ENREF_11)^.
4. IKDC: 6.3–16.7 points after cartilage repair[^12^](#_ENREF_12).

**eMethod 8: Meta-regression analysis**

If *I^2^* was ≥50, random effects meta-regression analysis was performed using the parameters selected *a priori* as follows: i) type of MSC treatment (0: injection, 1: implantation); ii) study design (0: non-RCT, 1: RCT); iii) risk of bias (Downs and Black score); iv) follow-up periods; v) baseline knee pain intensity or baseline physical function; vi) year of publication; vii) patient characteristics (mean age and %female sex); viii) rehabilitation (0: no, 1: yes); and ix) funding source (0: no, 1: yes). In this meta-regression analysis, we defined the presence of rehabilitation when patients were treated using physical therapy modalities, range of motion exercise, or muscle strength exercise at least one time after MSC treatment, and included is as independent variable. To examine the impact of bias risk on effects estimates, each item of the Downs and Black scale was also included in the meta-regression.

**eMethod 9: Quality assessment of evidence by using the GRADE approach**

The GRADE approach[^13^](#_ENREF_13) was used to evaluate the quality of the body of evidence for each primary outcome included in a meta-analysis. One reviewer (HI) graded the quality of the outcome measures of interest as high, moderate, low, and very low by using the following five domains: risk of bias, inconsistency, indirectness, imprecision, and publication bias[^13^](#_ENREF_13). The evidence quality was downgraded if i) primary outcomes have a high risk of bias; we defined this as a Downs and Black scale score of <8 for most of the trials in the meta-analysis; ii) heterogeneity between trials was more than substantial (*I^2^* ≥50%)[^1^](#_ENREF_1)^,^[^14^](#_ENREF_14); iii) the 95% CI of SMD was large; that is, clinical action differs if the 95% CI is the upper or lower boundary[^15^](#_ENREF_15); and iv) publication bias existed as evaluated with Egger’s regression test[^16^](#_ENREF_16) or undetermined because of the few included studies (<10 data set).

**eTable 1. Funding source**

| **Author** | **Funding** |
| --- | --- |
| ***Single-arm, prospective follow-up studies*** | |
| Bui, 2014 (Vietnam)[^17^](#_ENREF_17) | GeneWorld Ltd. |
| Centeno, 2008a (Unites states)[^18^](#_ENREF_18) | – |
| Centeno, 2008b (Unites states)[^19^](#_ENREF_19) | – |
| Davatchi, 2011 (Iran)[^20^](#_ENREF_20) | Tehran University of Medical Science |
| Davatchi, 2016 (Iran)[^21^](#_ENREF_21) | Tehran University of Medical Science |
| Emadedin, 2012 (Iran)[^22^](#_ENREF_22) | Royan Institute |
| Emadedin, 2015 (Iran)[^23^](#_ENREF_23) | Royan Institute |
| Fodor, 2016 (Unites states)[^24^](#_ENREF_24) | Aesthetic Surgery Education and Research Foundation (ASERF) |
| Kim, 2015c (Korea)[^25^](#_ENREF_25) | – |
| Kim, 2016 (Korea)[^26^](#_ENREF_26) | – |
| Koh, 2013 (Korea)[^27^](#_ENREF_27) | – |
| Koh, 2014a (Korea)[^28^](#_ENREF_28) | – |
| Koh, 2015 (Korea)[^29^](#_ENREF_29) | – |
| Michalek, 2015 (Czech Republic)[^30^](#_ENREF_30) | The Ministry of Education, Youth and Sports of the Czech Republic and European Union Operational Program Education for Competitiveness |
| Orozco, 2013 (Spain)[^31^](#_ENREF_31) | – |
| Orozco, 2014 (Spain)[^32^](#_ENREF_32) | – |
| Pak, 2011 (Korea)[^33^](#_ENREF_33) | – |
| Sampson, 2016 (Unites states)[^34^](#_ENREF_34) | – |
| Soler Rich, 2015 (Spain)[^35^](#_ENREF_35) |  |
| Soler, 2016 (Spain)[^36^](#_ENREF_36) | The Spanish Ministry of Science and Innovation. The Ministry of Economy and Competitiveness. European Regional Development Fund (ERDF) |
| Turajane, 2013 (Thailand)[^37^](#_ENREF_37) | THAISternLife Co., Ltd., Vejdusit Foundation, Surgeon in Chief Foundation, Police General Hospital, TRB Chermidica Co., Ltd. |

**eTable 1. (Continued)**

| **Author** | **Funding** |
| --- | --- |
| ***Quasi-experimental studies*** |  |
| Centeno, 2014 (Unites states)[^38^](#_ENREF_38) | – |
| Jo, 2014 (Korea)[^39^](#_ENREF_39) | Bio & Medical Technology Development Program and the Basic Science Research Program of the National Research Foundation (NRF) funded by the Korean government (MEST) and by K-STEM CELL |
| Kim, 2015a (Korea)[^40^](#_ENREF_40) | – |
| Kim, 2015b (Korea)[^41^](#_ENREF_41) | – |
| Koh, 2012 (Korea)[^42^](#_ENREF_42) | – |
| Nguyen, 2017 (Vietnam)[^43^](#_ENREF_43) | GeneWord Ltd. |
| Pers, 2016 (France)[^44^](#_ENREF_44) | The European Union Seventh Framework Programme. The Inserm Institute, the University of Montpellier. The Agence Nationale pour La Recherche |
|  |  |
| ***Randomized controlled trials*** |  |
| Gupta, 2016 (India)[^45^](#_ENREF_45) | Stempeutics Research Pvt. Ltd., Bangalore |
| Koh, 2014b (Korea)[^46^](#_ENREF_46) | – |
| Lamo-Espinosa, 2016 (Spain)[^47^](#_ENREF_47) | Instituto de Salud Carlos III |
| Varma, 2010 (India)[^48^](#_ENREF_48) | – |
| Vega, 2015 (Spain)[^49^](#_ENREF_49) | – |
| Wakitani, 2002 (Japan)[^50^](#_ENREF_50) | – |
| Wong, 2013 (Singapore)[^51^](#_ENREF_51) | – |

**eTable 2. Summary of rehabilitation programs**

Rehabilitation program frequently reported were weight-bearing schedule (13 [37.1%] studies)[^24-26^](#_ENREF_24)^,^[^28^](#_ENREF_28)^,^[^36^](#_ENREF_36)^,^[^38-41^](#_ENREF_38)^,^[^43^](#_ENREF_43)^,^[^46^](#_ENREF_46)^,^[^50^](#_ENREF_50)^,^[^51^](#_ENREF_51) and range of motion exercise (10 [28.6%] studies)[^25^](#_ENREF_25)^,^[^26^](#_ENREF_26)^,^[^28^](#_ENREF_28)^,^[^39-41^](#_ENREF_39)^,^[^43^](#_ENREF_43)^,^[^46^](#_ENREF_46)^,^[^50^](#_ENREF_50)^,^[^51^](#_ENREF_51). However, other information about rehabilitation programs such as muscle strength exercise (4 [11.4%] studies)[^39^](#_ENREF_39)^,^[^43^](#_ENREF_43)^,^[^46^](#_ENREF_46)^,^[^51^](#_ENREF_51), physical therapy modalities (3 [8.6%] studies)[^18^](#_ENREF_18)^,^[^27^](#_ENREF_27)^,^[^42^](#_ENREF_42), and pre-MSC treatment rehabilitation (1 [2.9%] studies)[^39^](#_ENREF_39) were poor.

| **Author** | **Pre-treatment Rehabilitation** | **Weight-bearing Schedule** | **Physical Therapy Modalities** | **Range of Motion Exercise** | **Muscle Strength Exercise** | **Sports Activity** | **Physical Therapist*** |
| --- | --- | --- | --- | --- | --- | --- | --- |
| ***Single-arm, prospective follow-up studies*** | | |  |  |  |  |  |
| Bui, 2014[^17^](#_ENREF_17) (Vietnam) | – | – | – | – | – | – | – |
| Centeno, 2008a[^18^](#_ENREF_18) (Unites states) | – | – | Ultrasound (medial aspect of knee, 20 min/day, 3 weeks) | – | – | – | – |
| Centeno, 2008b[^19^](#_ENREF_19) (Unites states) | – | – | – | – | – | – | X  (ROM measurement) |
| Davatchi, 2011[^20^](#_ENREF_20) (Iran) | – | – | – | – | – | – | – |
| Davatchi, 2016[^21^](#_ENREF_21) (Iran) | – | – | – | – | – | – | – |
| Emadedin, 2012[^22^](#_ENREF_22) (Iran) | – | – | – | – | – | – | – |
| Emadedin, 2015[^23^](#_ENREF_23) (Iran) | – | – | – | – | – | – | – |
| Fodor, 2016[^24^](#_ENREF_24) (Unites states) | – | ～Day 2 NWB with crutches | – | – | – | – | – |
| Kim, 2015c[^25^](#_ENREF_25) (Korea) | – | ～2 weeks NWB 2 weeks～ PWB 4 weeks～ FWB | – | ～2 weeks immobilized with knee brace 2 weeks～ active/passive movement | – | 3 months～ | – |
| Kim, 2016[^26^](#_ENREF_26) (Korea) | – | ～2 weeks NWB 2 weeks～ PWB 4 weeks～ FWB | – | ～2 weeks immobilized with knee brace 2 weeks～ active/passive movement | – | 3 months～ | – |
| Koh, 2013[^27^](#_ENREF_27) (Korea) | – | No restriction | Cold therapy | – | – | No restriction | – |
| Koh, 2014a[^28^](#_ENREF_28) (Korea) | – | ～2 weeks NWB 2 weeks～ PWB 4 weeks～ FWB | – | ～2 weeks immobilized with knee brace 2 weeks～ active/passive movement | – | 3 months～ | – |
| Koh, 2015[^29^](#_ENREF_29) (Korea) | – | – | – | – | – | – | – |
| Michalek, 2015[^30^](#_ENREF_30) (Czech Republic) | – | – | – | – | – | – | – |
| Orozco, 2013[^31^](#_ENREF_31) (Spain) | – | – | – | – | – | – | – |
| Orozco, 2014[^32^](#_ENREF_32) (Spain) | – | – | – | – | – | – | – |
| Pak, 2011[^33^](#_ENREF_33) (Korea) | – | No restriction | – | – | – | No restriction | – |
| Sampson, 2016[^34^](#_ENREF_34) (Unites states) | – | – | – | – | – | – | – |
| Soler Rich, 2015[^35^](#_ENREF_35) (Spain) | – | – | – | – | – | – | – |
| Soler, 2016[^36^](#_ENREF_36) (Spain) | – | ～Day 8 PWB with crutches Day 8～ FWB | – | – | – | – | – |
| Turajane, 2013[^37^](#_ENREF_37) (Thailand) | – | – | – | – | – | – | – |

**eTable 2. (Continued)**

| **Author** | **Pre-treatment Rehabilitation** | **Weight-bearing Schedule** | **Physical Therapy Modalities** | **Range of Motion Exercise** | **Muscle Strength Exercise** | **Sports Activity** | **Physical Therapist*** |
| --- | --- | --- | --- | --- | --- | --- | --- |
| ***Quasi-experimental studies*** | | |  |  |  |  |  |
| Centeno, 2014[^38^](#_ENREF_38) (Unites states) | – | ～6 weeks FWB with off-loader brace or patellar stabilizer brace | – | – | – | – | – |
| Jo, 2014[^39^](#_ENREF_39) (Korea) | Education (ROMex + quadriceps strengthening) | ～8 weeks TWB with crutch ～12 weeks stepwise increase in load bearing 12 weeks～ FWB | – | Day 1～ | Quadriceps strengthening (day 1～) | – | – |
| Kim, 2015a[^40^](#_ENREF_40) (Korea) | – | ～2 weeks NWB 2 weeks～ PWB 4 weeks～ FWB | – | ～2 weeks immobilized with knee brace 2 weeks～ active/passive movement | – | 3 months ～ | – |
| Kim, 2015b[^41^](#_ENREF_41) (Korea) | – | ～2 weeks NWB 2 weeks～ PWB 4 weeks～ FWB | – | ～2 weeks immobilized with knee brace 2 weeks ～ active/passive movement | – | 3 months ～ | – |
| Koh, 2012[^42^](#_ENREF_42) (Korea) | – | No restriction | Cold therapy |  | – | No restriction | – |
| Nguyen, 2017[^43^](#_ENREF_43) (Vietnam) | – | ～2 weeks NWB 2 weeks～ PWB 4 weeks～ FWB | – | Day 4～ CPM | Isometric quadriceps and hamstrings strengthening | Light sport activity: 6 month～; full sport activity: 12 months～ | – |
| Pers, 2016[^44^](#_ENREF_44) (France) | – | – | – | – | – | – | – |
| ***Randomized controlled trials*** | | |  |  |  |  |  |
| Gupta, 2016[^45^](#_ENREF_45) (India) | – | – | – | – | – | – | – |
| Koh, 2014b[^46^](#_ENREF_46) (Korea) | – | ～2 weeks TWB ～4weeks PWB 4 weeks～ FWB | – | 2 weeks～ knee flexion 0–90° | Isometric quadriceps strengthening, active ankle, and SLR exercises (day 1～) | – | – |
| Lamo-Espinosa, 2016[^47^](#_ENREF_47)  (Spain) | – | – | – | – | – | – | – |
| Varma, 2010[^48^](#_ENREF_48) (India) | – | – | – | – | – | – | – |
| Vega, 2015[^49^](#_ENREF_49) (Spain) | – | – | – | – | – | – | – |
| Wakitani, 2002[^50^](#_ENREF_50) (Japan) | – | 3～6 weeks PWB with crutches 8 weeks～ FWB | – | Day 4～ CPM | – | – | – |
| Wong, 2013[^51^](#_ENREF_51) (Singapore) | – | Day 2～6 weeks NWB with crutches | – | Day 2～4 weeks CPM (8 hour/day) | Stational bicycles, elliptical trainers, treadmill, and CKC exercise† | – | X†† |

CKC: Closed-kinetic Chain; CPM: Continuous Passive Motion; FWB: Full Weight Bearing; NWB: Non-weight Bearing; PWB: Partial Weight Bearing; ROM: Range of Motion; ROMex: Range of Motion Exercise; SLR: Straight Leg-raising; TWB: Toe-touch Weight Bearing.

* *X* indicates participation of physical therapist.

† Started after achieving full weight bearing and good ROM.

†† Patients who cannot undergo CPM received physical therapy training and be guided to complete 500 flexion and extension exercises per day.

**eTable 3. Downs and Black scale score of each study**

Inter-rater reliability was fair to good[^52^](#_ENREF_52) for all items (*κ* = 0.593, 95% CI: 0.515–0.670), for bias-related 7 items (*κ* = 0.636, 95% CI: 0.534–0.738), and confounding-related 6 items (*κ* = 0.473, 95% CI: 0.343–0.603).

| Author | Bias | | | | | | | |  | Confounding-Selection Bias | | | | | | Total score |
| --- | --- | --- | --- | --- | --- | --- | --- | --- | --- | --- | --- | --- | --- | --- | --- | --- |
|  | *14. Attempted to blind subjects to intervention* | *15. Attempt to blind assessors to intervention* | *16. Clarification for data dredging* | | *17. Adjusting for different lengths of follow-up* | *18. Appropriateness of statistical analyses* | *19. Compliance reliability of intervention* | *20. Valid and reliable main outcome measures* |  | *21. Recruitment from the same population* | *22. Recruitment over the same time period* | *23. Patients randomization to intervention group* | *24. Concealed randomization until recruitment complete* | *25. Adequate adjustment for confounders* | *26. Accounted for loss to follow-up* |  |
| ***Single-arm, prospective follow-up studies*** | | | | | |  |  |  |  |  |  |  |  |  |  |  |
| Bui, 2014[^17^](#_ENREF_17) | 0 | 0 | 1 | 1 | | 0 | 1 | 1 |  | 0 | 0 | 0 | 0 | 0 | 1 | 5 |
| Centeno, 2008a[^18^](#_ENREF_18) | 0 | 0 | 1 | NA* | | 0 | 1 | 1 |  | 0 | 0 | 0 | 0 | 0 | 1 | 4 |
| Centeno, 2008b[^19^](#_ENREF_19) | 0 | 0 | 1 | NA* | | 0 | 1 | 1 |  | 0 | 0 | 0 | 0 | 0 | 1 | 4 |
| Davatchi, 2011[^20^](#_ENREF_20) | 0 | 0 | 1 | 1 | | 0 | 1 | 0 |  | 0 | 0 | 0 | 0 | 0 | 1 | 4 |
| Davatchi, 2016[^21^](#_ENREF_21) | 0 | 0 | 1 | 0 | | 0 | 1 | 0 |  | 0 | 0 | 0 | 0 | 0 | 0 | 2 |
| Emardin, 2012[^22^](#_ENREF_22) | 0 | 0 | 1 | 1 | | 0 | 1 | 0 |  | 0 | 0 | 0 | 0 | 0 | 1 | 4 |
| Emardin, 2015[^23^](#_ENREF_23) | 0 | 0 | 1 | 1 | | 0 | 1 | 1 |  | 0 | 0 | 0 | 0 | 0 | 1 | 5 |
| Fodor, 2016[^24^](#_ENREF_24) | 0 | 0 | 1 | 1 | | 0 | 1 | 1 |  | 0 | 0 | 0 | 0 | 0 | 1 | 5 |
| Kim, 2015c[^25^](#_ENREF_25) | 0 | 0 | 1 | 1 | | 1 | 1 | 1 |  | 0 | 0 | 0 | 0 | 1 | 1 | 7 |
| Kim, 2016[^26^](#_ENREF_26) | 0 | 0 | 1 | 0 | | 1 | 1 | 1 |  | 0 | 1 | 0 | 0 | 1 | 1 | 7 |
| Koh, 2013[^27^](#_ENREF_27) | 0 | 0 | 1 | 1 | | 1 | 1 | 1 |  | 0 | 1 | 0 | 0 | 0 | 0 | 6 |
| Koh, 2014a[^28^](#_ENREF_28) | 0 | 0 | 1 | 1 | | 1 | 1 | 1 |  | 1 | 1 | 0 | 0 | 1 | 1 | 9 |
| Koh, 2015[^29^](#_ENREF_29) | 0 | 0 | 1 | 1 | | 1 | 1 | 1 |  | 0 | 1 | 0 | 0 | 1 | 0 | 7 |
| Michalek, 2015[^30^](#_ENREF_30) | 0 | 0 | 1 | 1 | | 1 | 1 | 1 |  | 0 | 1 | 0 | 0 | 0 | 1 | 7 |
| Orozco, 2013[^31^](#_ENREF_31) | 0 | 0 | 1 | 1 | | 0 | 1 | 1 |  | 0 | 1 | 0 | 0 | 0 | 1 | 6 |
| Orozco, 2014[^32^](#_ENREF_32) | 0 | 0 | 1 | 1 | | 0 | 1 | 1 |  | 0 | 0 | 0 | 0 | 0 | 1 | 5 |
| Pak, 2011[^33^](#_ENREF_33) | 0 | 0 | 1 | 1 | | 0 | 1 | 0 |  | 0 | 0 | 0 | 0 | 0 | 1 | 4 |
| Sampson, 2016[^34^](#_ENREF_34) | 0 | 0 | 1 | 0 | | 0 | 1 | 1 |  | 0 | 1 | 0 | 0 | 0 | 0 | 4 |
| Soler Rich, 2015[^35^](#_ENREF_35) | 0 | 0 | 1 | 1 | | 0 | 1 | 1 |  | 1 | 1 | 0 | 0 | 0 | 1 | 7 |
| Soler, 2016[^36^](#_ENREF_36) | 0 | 0 | 1 | 1 | | 0 | 1 | 1 |  | 1 | 1 | 0 | 0 | 0 | 1 | 7 |
| Turajane, 2013[^37^](#_ENREF_37) | 0 | 0 | 1 | 1 | | 0 | 1 | 1 |  | 0 | 1 | 0 | 0 | 0 | 1 | 6 |

**eTable 3. (Continued)**

| Author | Bias | | | | | | |  | Confounding-Selection Bias | | | | | | Total score |
| --- | --- | --- | --- | --- | --- | --- | --- | --- | --- | --- | --- | --- | --- | --- | --- |
|  | *14. Attempted to blind subjects to intervention* | *15. Attempt to blind assessors to intervention* | *16. Clarification for data dredging* | *17. Adjusting for different lengths of follow-up* | *18. Appropriateness of statistical analyses* | *19. Compliance reliability of intervention* | *20. Valid and reliable main outcome measures* |  | *21. Recruitment from the same population* | *22. Recruitment over the same time period* | *23. Patients randomization to intervention group* | *24. Concealed randomization until recruitment complete* | *25. Adequate adjustment for confounders* | *26. Accounted for loss to follow-up* |  |
| ***Quasi-experimental studies*** | | |  |  |  |  |  |  |  |  |  |  |  |  |  |
| Centeno, 2014[^38^](#_ENREF_38) | 0 | 0 | 0 | 1 | 1 | 1 | 1 |  | 0 | 1 | 0 | 0 | 1 | 0 | 6 |
| Jo, 2014[^39^](#_ENREF_39) | 0 | 0 | 1 | 1 | 1 | 1 | 1 |  | 1 | 0 | 0 | 0 | 1 | 0 | 7 |
| Kim, 2015a[^40^](#_ENREF_40) | 0 | 0 | 0 | 1 | 1 | 1 | 1 |  | 0 | 0 | 0 | 0 | 1 | 0 | 5 |
| Kim, 2015b[^41^](#_ENREF_41) | 0 | 0 | 1 | 1 | 0 | 1 | 1 |  | 0 | 0 | 0 | 0 | 1 | 0 | 5 |
| Koh, 2012[^42^](#_ENREF_42) | 0 | 0 | 1 | 1 | 1 | 1 | 1 |  | 0 | 1 | 0 | 0 | 0 | 1 | 7 |
| Nguyen, 2017[^43^](#_ENREF_43) | 0 | 0 | 1 | 1 | 0 | 1 | 1 |  | 0 | 1 | 0 | 0 | 1 | 1 | 7 |
| Pers, 2016[^44^](#_ENREF_44) | 0 | 0 | 1 | 1 | 0 | 1 | 1 |  | 1 | 1 | 0 | 0 | 0 | 1 | 7 |
|  |  |  |  |  |  |  |  |  |  |  |  |  |  |  |  |
| ***Randomized controlled trials*** | | |  |  |  |  |  |  |  |  |  |  |  |  |  |
| Gupta, 2016[^45^](#_ENREF_45) | 1 | 1 | 1 | 1 | 0 | 1 | 1 |  | 1 | 1 | 1 | 1 | 1 | 1 | 12 |
| Koh, 2014b[^46^](#_ENREF_46) | 0 | 0 | 0 | 1 | 1 | 1 | 1 |  | 0 | 1 | 1 | 0 | 1 | 0 | 7 |
| Lamo-Espinosa, 2016[^47^](#_ENREF_47) | 0 | 0 | 1 | 1 | 0 | 1 | 0 |  | 1 | 1 | 1 | 0 | 1 | 1 | 8 |
| Varma, 2010[^48^](#_ENREF_48) | 0 | 0 | 1 | 1 | 0 | 1 | 1 |  | 1 | 0 | 0 | 0 | 0 | 1 | 6 |
| Vega, 2015[^49^](#_ENREF_49) | 1 | 1 | 1 | 1 | 0 | 1 | 1 |  | 1 | 1 | 1 | 1 | 1 | 1 | 12 |
| Wakitani, 2002[^50^](#_ENREF_50) | 0 | 0 | 0 | 1 | 1 | 1 | 0 |  | 0 | 0 | 0 | 0 | 0 | 0 | 3 |
| Wong, 2013[^51^](#_ENREF_51) | 0 | 0 | 1 | 1 | 1 | 1 | 0 |  | 0 | 0 | 1 | 0 | 1 | 1 | 7 |

* Not applicable (NA) due to a single case report. All items were scored 1 for fulfilling the criterion or 0 if the criterion were not filled. Publication that did not provide sufficient details to fulfill the criterion were also given a 0 for unable to be determined in accordance of the original index of Downs and Black scale[^6^](#_ENREF_6).

**eTable 4. Meta-regression analysis of study characteristics on the effects estimate for the VAS pain score**

A higher score of the Downs and Black scale (i.e., low risk of bias) was a significant factor associated with a higher (i.e., lower effect) SMD.

| Variables | Regression Coefficient (95% CI) | *P*-value |
| --- | --- | --- |
| Type of treatment (0: injection, 1: implantation) | 0.017 (-3.693, 3.728) | 0.992 |
| Study design (0: non-RCT, 1: RCT) | 1.409 (-0.759, 3.578) | 0.191 |
| **Downs and Black scale score, per point** | **0.415 (0.042, 0.789)** | **0.031** |
| Follow-up periods, per month | -0.017 (-0.098, 0.064) | 0.665 |
| Baseline knee pain intensity, per mm | -0.059 (-0.144, 0.026) | 0.163 |
| Year of publication, per year | 0.292 (-0.464, 1.047) | 0.432 |
| Age, per year | 0.005 (-0.047, 0.057) | 0.836 |
| %Female sex, per percent | -0.001 (-0.023, 0.022) | 0.942 |
| Rehabilitation (0: no, 1: yes) | 0.451 (-1.909, 2.811) | 0.696 |
| Founding source (0: no, 1: yes) | -0.555 (-2.911, 1.801) | 0.630 |

Bold type represents a statistically significant result. A positive value for regression coefficient means unfavorable of MSC treatment.

**eTable 5. Meta-regression analysis estimating the impact of each risks of bias in the Downs and Black scale on the effects estimate for the VAS pain score**

Clear patients’ recruitment site was a significant factor associated with a higher (i.e., lower effect) SMD.

| Downs and Black Scale (0: 0 point [bias risk, yes], 1: 1 point [bias risk, no]) | Frequency, no (%) | | Regression Coefficient (95% CI) | *P*-value |
| --- | --- | --- | --- | --- |
|  | Bias risk, no | Bias risk, yes |  |  |
| 14. Attempted to blind subjects to intervention | 5 (18.5) | 22 (81.5) | 1.858 (-0.529, 4.246) | 0.121 |
| 15. Attempt to blind assessors to intervention | 5 (18.5) | 22 (81.5) | 1.858 (-0.529, 4.246) | 0.121 |
| 16. Clarification for data dredging | 0 (0.0) | 27 (100.0) | NA | NA |
| 17. Adjusting for different lengths of follow-up | 24 (88.9) | 3 (11.1) | 1.063 (-4.048, 6.174) | 0.670 |
| 18. Appropriateness of statistical analyses | 6 (22.2) | 21 (77.8) | 0.337 (-2.026, 2.701) | 0.770 |
| 19. Compliance reliability of intervention | 27 (100.0) | 0 (0.0) | NA | NA |
| 20. Valid and reliable main outcome measures | 22 (81.5) | 5 (18.5) | 0.332 (-2.416, 3.079) | 0.805 |
| **21. Recruitment from the same population** | **15 (55.6)** | **12 (44.4)** | **2.012 (0.090, 3.935)** | **0.041** |
| 22. Recruitment over the same time period | 17 (63.0) | 10 (37.0) | 2.007 (-0.067, 4.081) | 0.057 |
| 23. Patients randomization to intervention group | 7 (25.9) | 20 (74.1) | 1.409 (-0.759, 3.578) | 0.191 |
| 24. Concealed randomization until recruitment complete | 5 (18.5) | 22 (81.5) | 1.858 (-0.529, 4.246) | 0.121 |
| 25. Adequate adjustment for confounders | 12 (44.4) | 15 (55.6) | 1.454 (-0.494, 3.402) | 0.136 |
| 26. Accounted for loss to follow-up | 21 (77.8) | 6 (22.2) | -0.042 (-2.410, 2.327) | 0.971 |

NA: Not applicable. Bold type represents a statistically significant result. A positive value for regression coefficient means unfavorable of MSC treatment.

**eTable 6. Meta-regression analysis of study characteristics on the effects estimate for the VAS pain score in 3 RCTs with 7 data sets**

A higher score of the Downs and Black scale (i.e., low risk of bias) and younger age were significant factors associated with higher (i.e., lower effect) SMDs.

| Variables | Regression Coefficient (95% CI) | *P*-value |
| --- | --- | --- |
| Type of treatment (0: injection, 1: implantation) | NA | NA |
| **Downs and Black scale score, per point** | **0.414 (0.097, 0.731)** | **0.020** |
| Follow-up periods, per month | NA | NA |
| Baseline knee pain intensity, per mm | -0.041 (-0.152, 0.071) | 0.391 |
| Year of publication, per year | 0.290 (-0.478, 1.058) | 0.941 |
| **Age, per year** | **-0.228 (-0.348, -0.108)** | **0.005** |
| %Female sex, per percent | 0.016 (-0.034, 0.066) | 0.445 |
| Rehabilitation (0: no, 1: yes) | NA | NA |
| Founding source (0: no, 1: yes) | 0.090 (-2.860, 3.040) | 0.941 |

NA: Not applicable. Bold type represents a statistically significant result. A positive value for regression coefficient means unfavorable of MSC treatment.

**eTable 7. Meta-regression analysis estimating the impact of each risks of bias in the Downs and Black scale on the effects estimate for the VAS pain score in 3 RCTs with 7 data sets**

Blinding of participants and assessors, valid and reliable outcome measures, and concealed allocation were significant factors associated with higher (i.e., lower effect) SMDs.

| Downs and Black Scale (0: 0 point [bias risk, yes], 1: 1 point [bias risk, no]) | Frequency, no (%) | | Regression Coefficient (95% CI) | *P*-value |
| --- | --- | --- | --- | --- |
|  | Bias risk, no | Bias risk, yes |  |  |
| **14. Attempted to blind subjects to intervention** | 5 (71.4) | 2 (28.6) | **1.655 (0.386, 2.924)** | **0.020** |
| **15. Attempt to blind assessors to intervention** | 5 (71.4) | 2 (28.6) | **1.655 (0.386, 2.924)** | **0.020** |
| 16. Clarification for data dredging | 0 (0.0) | 7 (100.0) | NA | NA |
| 17. Adjusting for different lengths of follow-up | 0 (0.0) | 7 (100.0) | NA | NA |
| 18. Appropriateness of statistical analyses | 0 (0.0) | 7 (100.0) | NA | NA |
| 19. Compliance reliability of intervention | 0 (0.0) | 7 (100.0) | NA | NA |
| **20. Valid and reliable main outcome measures** | 5 (71.4) | 2 (28.6) | **1.655 (0.386, 2.924)** | **0.020** |
| 21. Recruitment from the same population | 7 (100.0) | 0 (0.0) | NA | NA |
| 22. Recruitment over the same time period | 7 (100.0) | 0 (0.0) | NA | NA |
| 23. Patients randomization to intervention group | 7 (100.0) | 0 (0.0) | NA | NA |
| **24. Concealed randomization until recruitment complete** | 5 (71.4) | 2 (28.6) | **1.655 (0.386, 2.924)** | **0.020** |
| 25. Adequate adjustment for confounders | 7 (100.0) | 0 (0.0) | NA | NA |
| 26. Accounted for loss to follow-up | 7 (100.0) | 0 (0.0) | NA | NA |

NA: Not applicable. Bold type represents a statistically significant result. A positive value for regression coefficient means unfavorable of MSC treatment.

**eTable 8. Meta-regression analysis of study characteristics on the effects estimate for the self-reported functional measures**

Implantation technique (compared to injection), lower Downs and Black scale score, presence of rehabilitation, and absence of funding source were significant factors associated with higher (i.e., higher effect) SMDs. Notably, presence of rehabilitation was a significant effect modifier of SMD.

| Variables | Regression Coefficient (95% CI) | *P*-value |
| --- | --- | --- |
| **Type of treatment (0: injection, 1: implantation)** | **1.556 (0.708, 2.404)** | **<0.001** |
| Study design (0: non-RCT, 1: RCT) | -0.937 (-1.917, 0.042) | 0.060 |
| **Downs and Black scale score, per point** | **-0.268 (-0.446, -0.091)** | **0.005** |
| Follow-up periods, per month | 0.039 (-0.001, 0.079) | 0.058 |
| Baseline functional score, per point | 0.019 (-0.012, 0.051) | 0.210 |
| Year of publication, per year | -0.306 (-0.640, 0.028) | 0.071 |
| Age, per year | -0.024 (-0.122, 0.074) | 0.622 |
| %Female sex, per percent | 0.000 (-0.027, 0.027) | 0.988 |
| **Rehabilitation (0: no, 1: yes)** | **0.881 (0.049, 1.712)** | **0.039** |
| **Founding source (0: no, 1: yes)** | **-0.925 (-1.758, -0.092)** | **0.031** |

Bold type represents a statistically significant result. A positive value for regression coefficient means favorable of MSC treatment.

**eTable 9. Meta-regression analysis estimating the impact of each risks of bias in the Downs and Black scale on the effects estimate for the self-reported functional measures**

Blinding of participants, unblinding of assessors, unclear patients’ recruitment site, non-randomization and non-concealed allocation were significant factors associated with higher (i.e., higher effect) SMDs.

| Downs and Black Scale (0: 0 point [bias risk, yes], 1: 1 point [bias risk, no]) | Frequency, no (%) | | Regression Coefficient (95% CI) | *P*-value |
| --- | --- | --- | --- | --- |
|  | Bias risk, no | Bias risk, yes |  |  |
| **14. Attempted to blind subjects to intervention** | **4 (13.8)** | **25 (86.2)** | **5.480 (4.273, 6.687)** | **<0.001** |
| **15. Attempt to blind assessors to intervention** | **4 (13.8)** | **25 (86.2)** | **-1.509 (-2.664, -0.354)** | **0.012** |
| 16. Clarification for data dredging | 27 (93.1) | 2 (6.9) | -1.223 (-2.925, 0.478) | 0.152 |
| 17. Adjusting for different lengths of follow-up | 28 (96.6) | 1 (3.4) | -1.439 (-3.828, 0.951) | 0.227 |
| 18. Appropriateness of statistical analyses | 11 (37.9) | 18 (62.1) | 0.814 (-0.052, 1.680) | 0.064 |
| 19. Compliance reliability of intervention | 29 (100.0) | 0 (0.0) | NA | NA |
| 20. Valid and reliable main outcome measures | 26 (89.7) | 3 (10.3) | 0.228 (-1.241, 1.700) | 0.753 |
| **21. Recruitment from the same population** | **15 (51.7)** | **14 (48.3)** | **-1.442 (-2.135, -0.750)** | **<0.001** |
| 22. Recruitment over the same time period | 19 (65.5) | 10 (34.5) | -0.610 (-1.522, 0.301) | 0.181 |
| **23. Patients randomization to intervention group** | **6 (20.7)** | **23 (79.3)** | **-1.254 (-2.243, -0.264)** | **0.015** |
| **24. Concealed randomization until recruitment complete** | **4 (13.8)** | **25 (86.2)** | **-1.509 (-2.664, 0.354)** | **0.012** |
| 25. Adequate adjustment for confounders | 18 (62.1) | 11 (37.9) | -0.140 (-1.062, 0.781) | 0.757 |
| 26. Accounted for loss to follow-up | 20 (69.0) | 9 (31.0) | -0.318 (-1.278, 0.643) | 0.503 |

NA: Not applicable. Bold type represents a statistically significant result. A positive value for regression coefficient means favorable of MSC treatment.

**eTable 10. Meta-regression analysis of study characteristics on the effects estimate for the cartilage quality**

Presence of funding source was a significant factor associated with a higher (i.e., lower effect) SMD.

| Variables | Regression Coefficient (95% CI) | *P*-value |
| --- | --- | --- |
| Type of treatment (0: injection, 1: implantation) | NA | NA |
| Study design (0: non-RCT, 1: RCT) | -1.348 (-6.728, 4.032) | 0.525 |
| Downs and Black scale score, per point | -0.134 (-1.108, 0.840) | 0.722 |
| Follow-up periods, per month | NA | NA |
| Year of publication, per year | 0.008 (-0.136, 0.152) | 0.881 |
| Age, per year | 0.111 (-0.188, 0.411) | 0.361 |
| %Female sex, per percent | 0.103 (-0.092, 0.299) | 0.215 |
| Rehabilitation (0: no, 1: yes) | NA | NA |
| **Founding source (0: no, 1: yes)** | **2.920 (1.658, 4.182)** | **0.003** |

NA: Not applicable. Bold type represents a statistically significant result. A positive value for regression coefficient means unfavorable of MSC treatment.

**eFigure 1. Review flow diagram**

The database search yielded 659 studies. After adjusting for duplicated studies, titles and abstracts of 512 studies were screened, and the remaining 73 studies were assessed for eligibility by full-text screening. Percent agreement of removing duplicated studies was 96.6%. Inter-rater reliability was fair to good[^52^](#_ENREF_52) for determining eligibility from title and abstract (*κ* = 0.561, 95% CI: 0.432–0.689), and excellent[^52^](#_ENREF_52) for that from full-text studies (*κ* = 0.930, 95% CI: 0.837–1.000). Exclusion reasons for the 42 studies during full-text screening were: publication in a non-peer reviewed journal (1; 2.4%), letter or review (24; 57.1%), patients with non-tibiofemoral joint OA (10; 23.8%), and treatment strategy without MSCs (7; 16.7%). Finally, thirty-one studies met the eligibility criteria. Furthermore, citation index was performed for the final 31 included studies and 4 additional studies were included in accordance with pre-specified inclusion criteria provided in eMethod 1; in total, 35 studies were used in the meta-analysis.


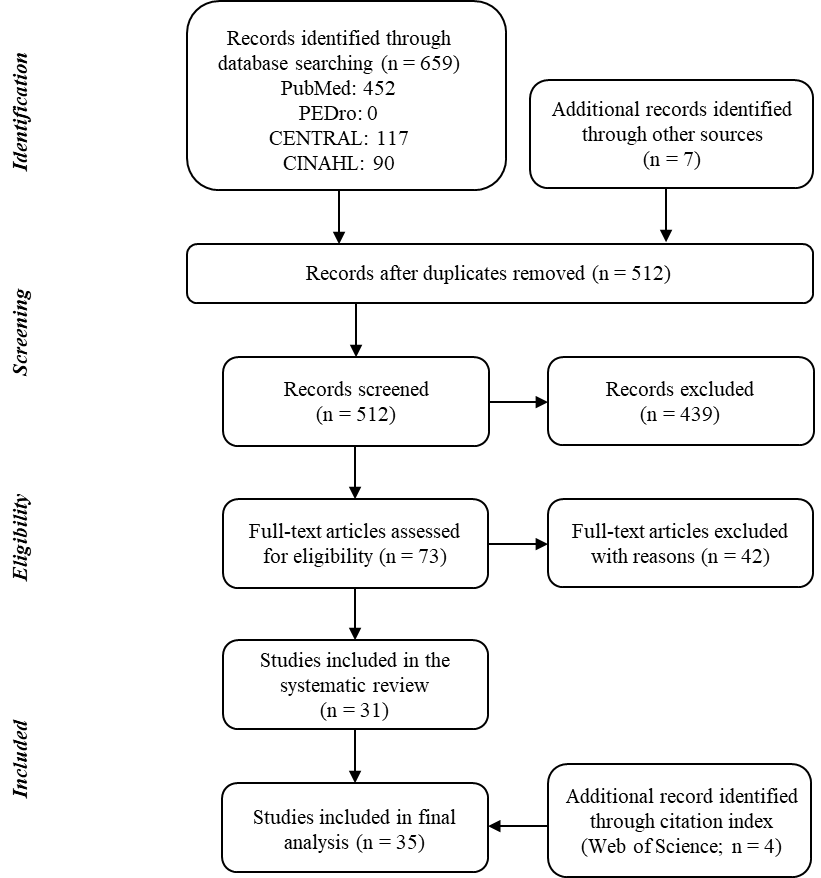


**eFigure 2. Funnel plot representing publication bias across 19 studies with 27 data sets shows a comparison of the effects of MSC treatment on VAS knee pain score**

Egger’s regression test was positive (*P* = 0.016), suggesting a presence of publication bias. Using the trim-and-fill method[^53^](#_ENREF_53), the intervention effect is adjusted for possible missing 6 articles (filled black circles) amongst published articles (open circles). The vertical line represents adjusted pooled SMD and 2 diagonal lines represent pseudo 95% confidence limits around the adjusted pooled SMD for each standard error on the vertical axis. The white and black diamonds represent the observed and adjusted effect size, respectively. Note that 3 data set from 3 single-arm prospective follow-up studies[^18^](#_ENREF_18)^,^[^19^](#_ENREF_19)^,^[^33^](#_ENREF_33) cannot be provided here because of a small sample size (n = 1 or 2).

**
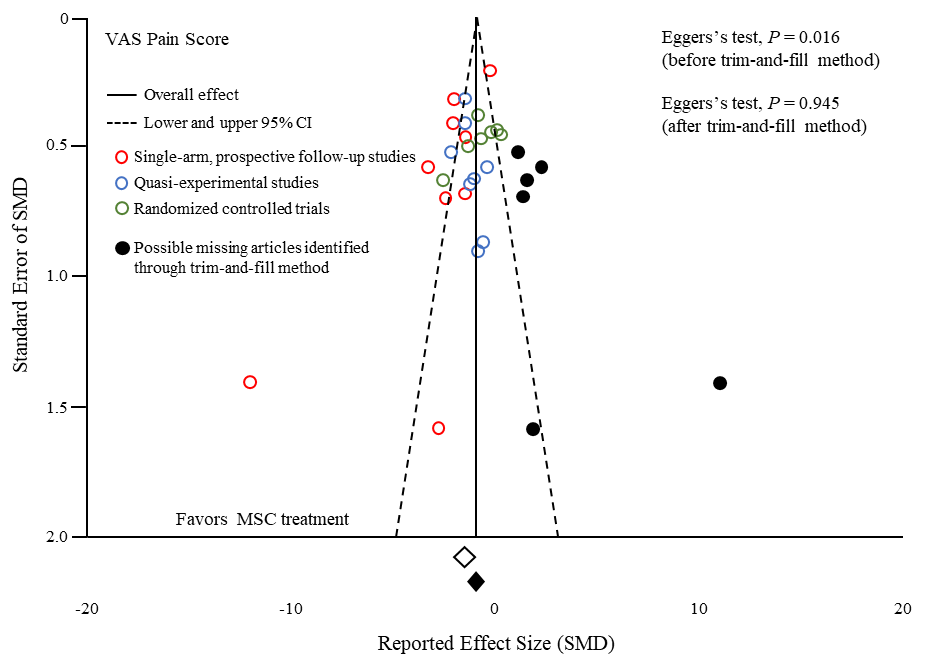
**

**eFigure 3. Funnel plot representing publication bias across 3 RCTs with 7 data sets shows a comparison of the effects of MSC treatment on VAS knee pain score**

The vertical line represents pooled SMD and 2 diagonal lines represent pseudo 95% confidence limits around the pooled SMD for each standard error on the vertical axis. No small-study effect was visually observed by two independent reviewers.

**
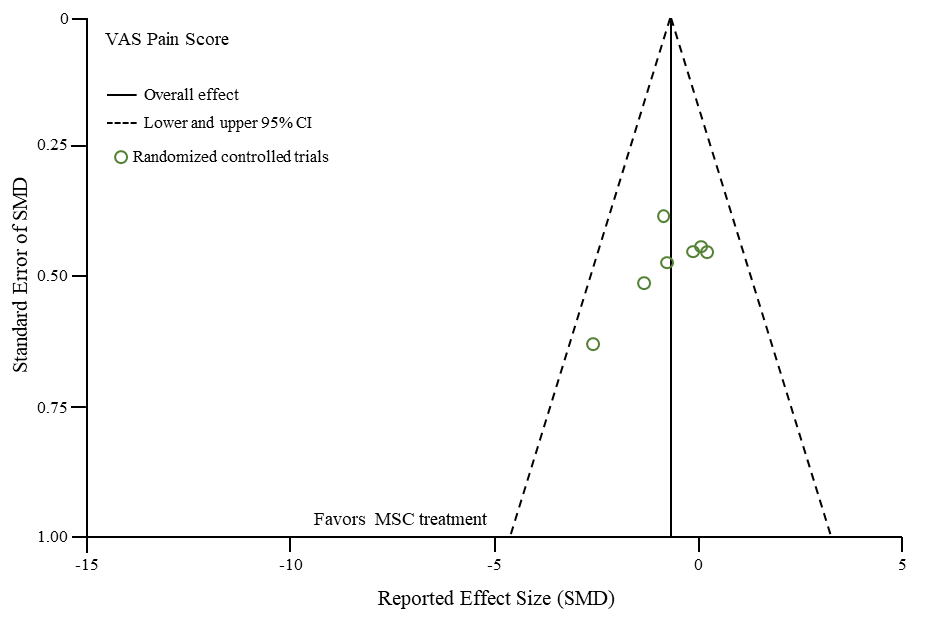
**

**eFigure 4. Funnel plot representing publication bias across 19 studies with 29 data sets shows a comparison of the effects of MSC treatment on self-reported physical function**

The vertical line represents pooled SMD and 2 diagonal lines represent pseudo 95% confidence limits around the pooled SMD for each standard error on the vertical axis. Egger’s regression test was negative (*P* = 0.516), suggesting an absence of significant publication bias.

**
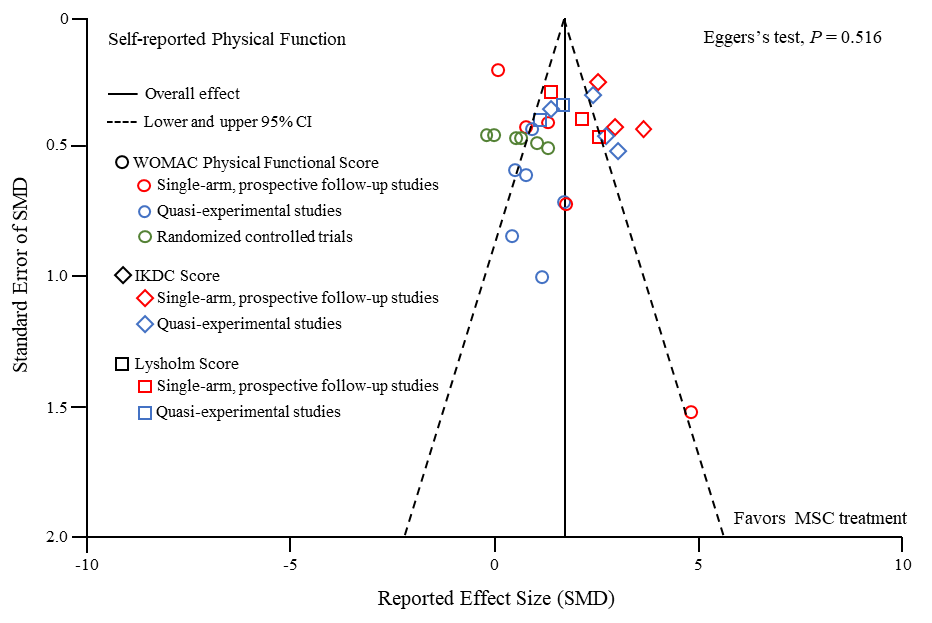
**

**eFigure 5. Funnel plot representing publication bias across 2 RCTs with 6 data sets shows a comparison of the effects of MSC treatment on self-reported physical function**

The vertical line represents pooled SMD and 2 diagonal lines represent pseudo 95% confidence limits around the pooled SMD for each standard error on the vertical axis. No small-study effect was visually observed by two independent reviewers.

**
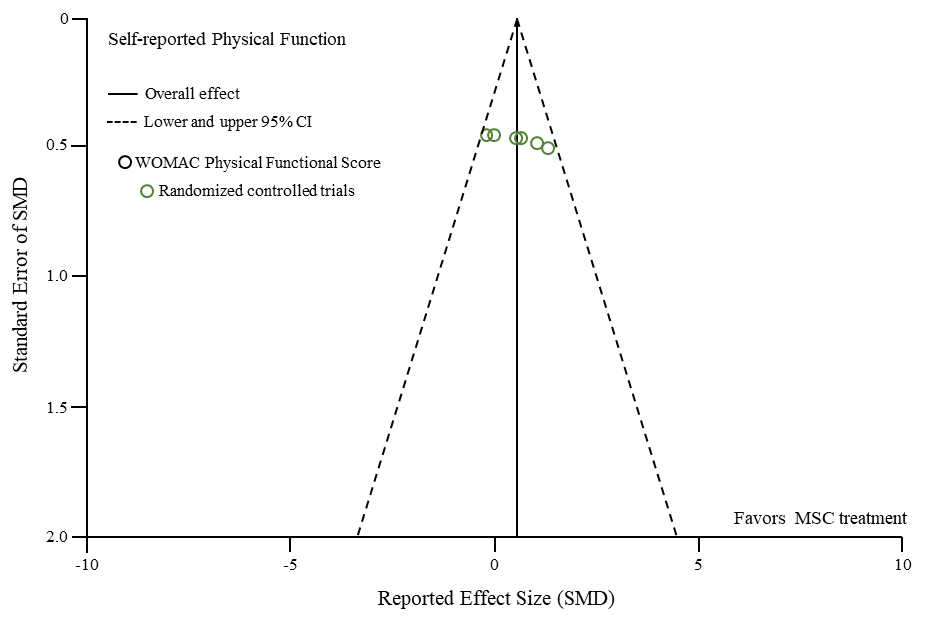
**

**eFigure 6. Funnel plot representing publication bias across 2 studies with 4 data sets shows a comparison of the effects of MSC treatment on cartilage volume**

The vertical line represents pooled SMD and 2 diagonal lines represent pseudo 95% confidence limits around the pooled SMD for each standard error on the vertical axis. No small-study effect was visually observed by two independent reviewers.

**
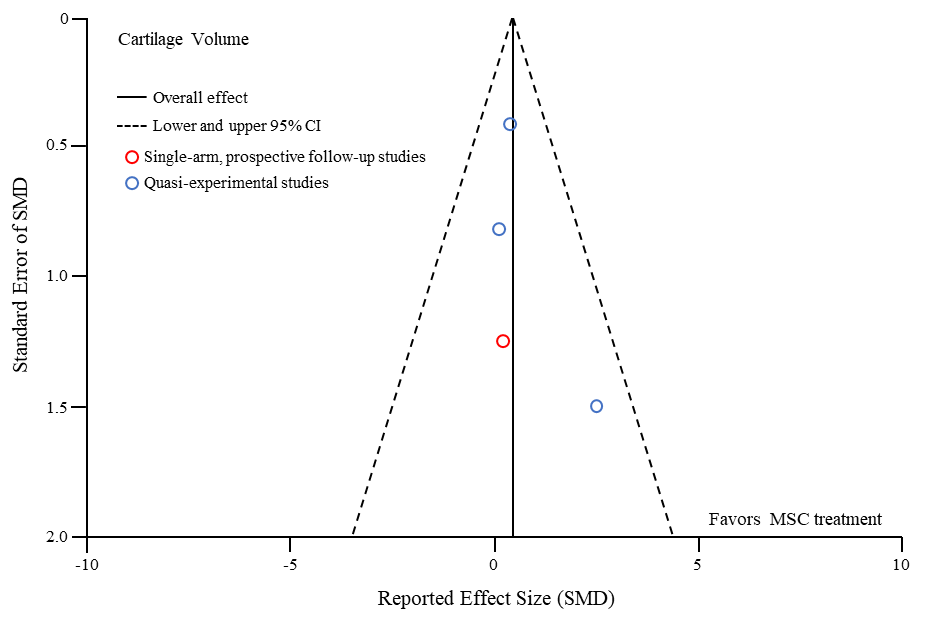
**

**eFigure 7. Funnel plot representing publication bias across 5 studies with 7 data sets shows a comparison of the effects of MSC treatment on cartilage quality**

The vertical line represents pooled SMD and 2 diagonal lines represent pseudo 95% confidence limits around the pooled SMD for each standard error on the vertical axis. No small-study effect was visually observed by two independent reviewers. Note that 1 data set from quasi-experimental study[^44^](#_ENREF_44) cannot be provided here because of a small sample size (n = 1).

**
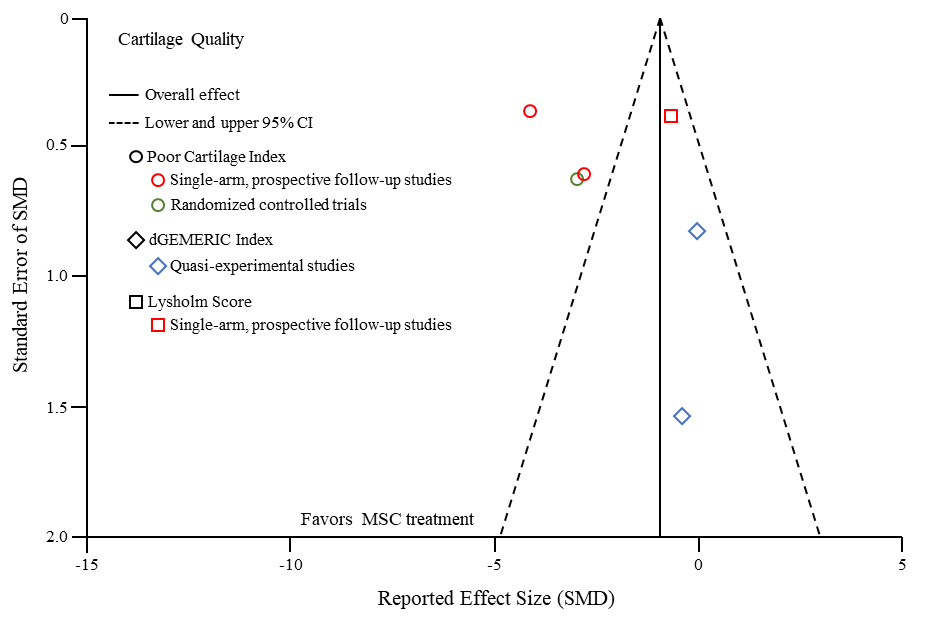
**

**eFigure 8. Adverse event rate and 95%CI in each included study**

The vertical line at 0 represents no adverse event. Of 35 studies, 17 (48.6%) reported adverse events related to MSC treatment. Adverse event included knee pain or swelling. Minor adverse events (knee pain or swelling) were reported with a wide-ranging prevalence of 2–60%.


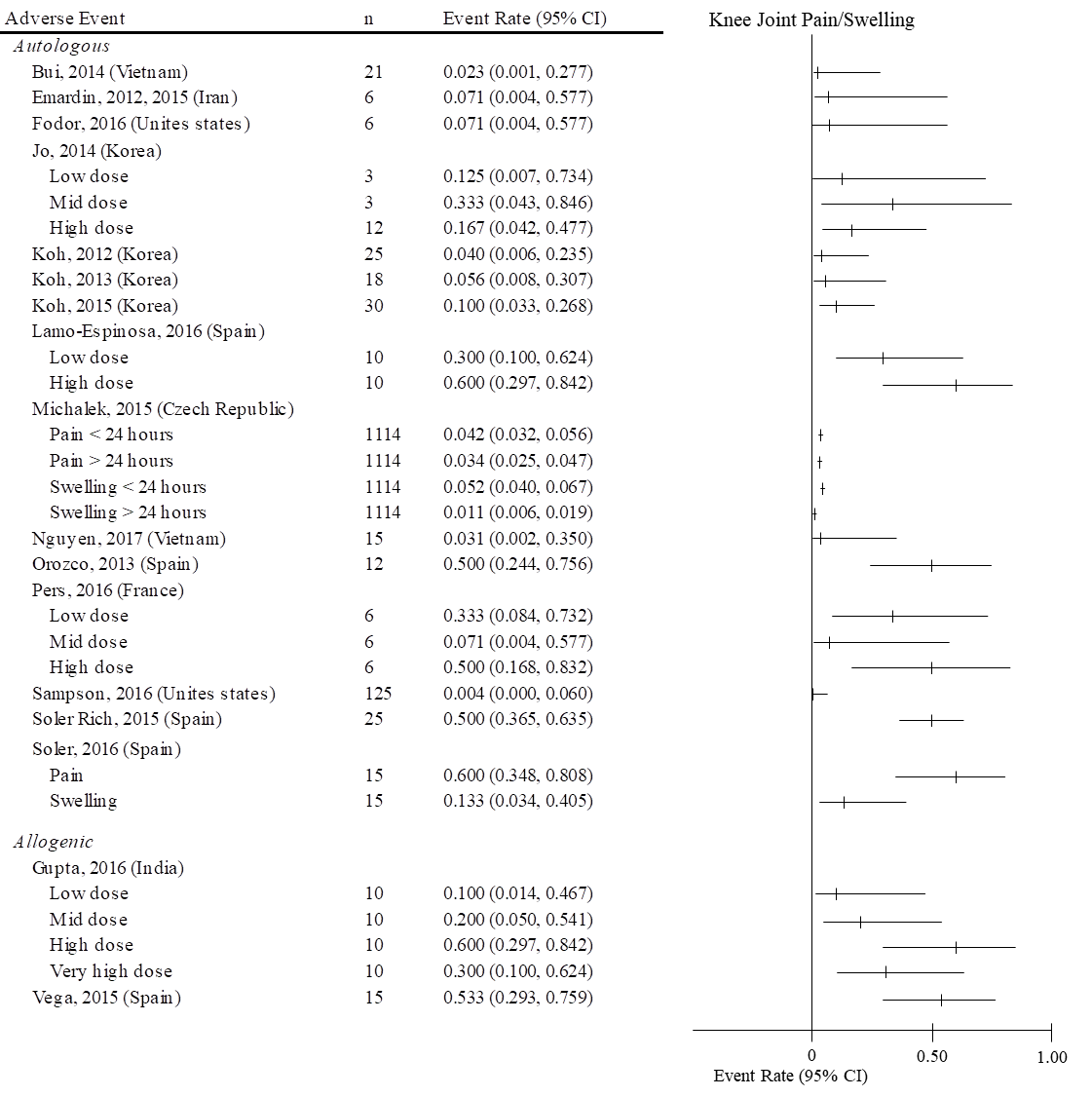


**Supplementary References**

1. Higgins, J.P. & Green, S. *Cochrane handbook for systematic reviews of interventions*, (John Wiley & Sons, 2011).

2. Altman, R.*, et al.* Development of criteria for the classification and reporting of osteoarthritis. Classification of osteoarthritis of the knee. Diagnostic and Therapeutic Criteria Committee of the American Rheumatism Association. *Arthritis Rheum* **29**, 1039-1049 (1986).

3. Hunter, D.J.*, et al.* Imaging biomarker validation and qualification report: sixth OARSI Workshop on Imaging in Osteoarthritis combined with third OA Biomarkers Workshop. *Osteoarthritis Cartilage* **21**, 939-942 (2013).

4. Guermazi, A., Eckstein, F., Hunter, D. & Roemer, F. 7th International Workshop on Osteoarthritis Imaging report: "imaging in OA--now is the time to move ahead". *Osteoarthritis Cartilage* **23**, 888-896 (2015).

5. Buck, R.J.*, et al.* Osteoarthritis may not be a one-way-road of cartilage loss--comparison of spatial patterns of cartilage change between osteoarthritic and healthy knees. *Osteoarthritis Cartilage* **18**, 329-335 (2010).

6. Downs, S.H. & Black, N. The feasibility of creating a checklist for the assessment of the methodological quality both of randomised and non-randomised studies of health care interventions. *Journal of epidemiology and community health* **52**, 377-384 (1998).

7. Sanderson, S., Tatt, I.D. & Higgins, J.P. Tools for assessing quality and susceptibility to bias in observational studies in epidemiology: a systematic review and annotated bibliography. *Int J Epidemiol* **36**, 666-676 (2007).

8. Deeks, J.J. & Higgins, J.P. Statistical algorithms in review manager 5. *Statistical Methods Group of The Cochrane Collaboration*, 1-11 (2010).

9. Wiebe, N.*, et al.* A systematic review identifies a lack of standardization in methods for handling missing variance data. *J Clin Epidemiol* **59**, 342-353 (2006).

10. Tubach, F.*, et al.* Evaluation of clinically relevant changes in patient reported outcomes in knee and hip osteoarthritis: the minimal clinically important improvement. *Ann Rheum Dis* **64**, 29-33 (2005).

11. Collins, N.J., Misra, D., Felson, D.T., Crossley, K.M. & Roos, E.M. Measures of knee function: International Knee Documentation Committee (IKDC) Subjective Knee Evaluation Form, Knee Injury and Osteoarthritis Outcome Score (KOOS), Knee Injury and Osteoarthritis Outcome Score Physical Function Short Form (KOOS-PS), Knee Outcome Survey Activities of Daily Living Scale (KOS-ADL), Lysholm Knee Scoring Scale, Oxford Knee Score (OKS), Western Ontario and McMaster Universities Osteoarthritis Index (WOMAC), Activity Rating Scale (ARS), and Tegner Activity Score (TAS). *Arthritis care & research* **63 Suppl 11**, S208-228 (2011).

12. Greco, N.J.*, et al.* Responsiveness of the International Knee Documentation Committee Subjective Knee Form in comparison to the Western Ontario and McMaster Universities Osteoarthritis Index, modified Cincinnati Knee Rating System, and Short Form 36 in patients with focal articular cartilage defects. *Am J Sports Med* **38**, 891-902 (2010).

13. Balshem, H.*, et al.* GRADE guidelines: 3. Rating the quality of evidence. *J Clin Epidemiol* **64**, 401-406 (2011).

14. Guyatt, G.H.*, et al.* GRADE guidelines: 7. Rating the quality of evidence--inconsistency. *J Clin Epidemiol* **64**, 1294-1302 (2011).

15. Guyatt, G.H.*, et al.* GRADE guidelines 6. Rating the quality of evidence--imprecision. *J Clin Epidemiol* **64**, 1283-1293 (2011).

16. Guyatt, G.H.*, et al.* GRADE guidelines: 5. Rating the quality of evidence--publication bias. *J Clin Epidemiol* **64**, 1277-1282 (2011).

17. Bui, K.H.-T.*, et al.* Symptomatic knee osteoarthritis treatment using autologous adipose derived stem cells and platelet-rich plasma: a clinical study. *Biomedical Research and Therapy* **1**, 02-08 (2014).

18. Centeno, C.J.*, et al.* Regeneration of meniscus cartilage in a knee treated with percutaneously implanted autologous mesenchymal stem cells. *Med Hypotheses* **71**, 900-908 (2008).

19. Centeno, C.J.*, et al.* Increased knee cartilage volume in degenerative joint disease using percutaneously implanted, autologous mesenchymal stem cells. *Pain physician* **11**, 343-353 (2008).

20. Davatchi, F., Abdollahi, B.S., Mohyeddin, M., Shahram, F. & Nikbin, B. Mesenchymal stem cell therapy for knee osteoarthritis. Preliminary report of four patients. *Int J Rheum Dis* **14**, 211-215 (2011).

21. Davatchi, F., Sadeghi Abdollahi, B., Mohyeddin, M. & Nikbin, B. Mesenchymal stem cell therapy for knee osteoarthritis: 5 years follow-up of three patients. *Int J Rheum Dis* **19**, 219-225 (2016).

22. Emadedin, M.*, et al.* Intra-articular injection of autologous mesenchymal stem cells in six patients with knee osteoarthritis. *Arch Iran Med* **15**, 422-428 (2012).

23. Emadedin, M.*, et al.* Long-Term Follow-up of Intra-articular Injection of Autologous Mesenchymal Stem Cells in Patients with Knee, Ankle, or Hip Osteoarthritis. *Arch Iran Med* **18**, 336-344 (2015).

24. Fodor, P.B. & Paulseth, S.G. Adipose Derived Stromal Cell (ADSC) Injections for Pain Management of Osteoarthritis in the Human Knee Joint. *Aesthet Surg J* **36**, 229-236 (2016).

25. Kim, Y.S., Choi, Y.J. & Koh, Y.G. Mesenchymal stem cell implantation in knee osteoarthritis: an assessment of the factors influencing clinical outcomes. *The American journal of sports medicine* **43**, 2293-2301 (2015).

26. Kim, Y.S.*, et al.* Assessment of clinical and MRI outcomes after mesenchymal stem cell implantation in patients with knee osteoarthritis: a prospective study. *Osteoarthritis and cartilage* **24**, 237-245 (2016).

27. Koh, Y.G.*, et al.* Mesenchymal stem cell injections improve symptoms of knee osteoarthritis. *Arthroscopy* **29**, 748-755 (2013).

28. Koh, Y.G., Choi, Y.J., Kwon, O.R. & Kim, Y.S. Second-Look Arthroscopic Evaluation of Cartilage Lesions After Mesenchymal Stem Cell Implantation in Osteoarthritic Knees. *The American journal of sports medicine* **42**, 1628-1637 (2014).

29. Koh, Y.G., Choi, Y.J., Kwon, S.K., Kim, Y.S. & Yeo, J.E. Clinical results and second-look arthroscopic findings after treatment with adipose-derived stem cells for knee osteoarthritis. *Knee surgery, sports traumatology, arthroscopy : official journal of the ESSKA* **23**, 1308-1316 (2015).

30. Michalek, J.*, et al.* WITHDRAWN: Autologous adipose tissue-derived stromal vascular fraction cells application in patients with osteoarthritis. *Cell Transplant* (2015).

31. Orozco, L.*, et al.* Treatment of knee osteoarthritis with autologous mesenchymal stem cells: a pilot study. *Transplantation* **95**, 1535-1541 (2013).

32. Orozco, L.*, et al.* Treatment of knee osteoarthritis with autologous mesenchymal stem cells: two-year follow-up results. *Transplantation* **97**, e66-68 (2014).

33. Pak, J. Regeneration of human bones in hip osteonecrosis and human cartilage in knee osteoarthritis with autologous adipose-tissue-derived stem cells: a case series. *J Med Case Rep* **5**, 296 (2011).

34. Sampson, S.*, et al.* Intra-articular bone marrow concentrate injection protocol: short-term efficacy in osteoarthritis. *Regen Med* **11**, 511-520 (2016).

35. Soler Rich, R.*, et al.* Treatment of knee osteoarthritis with autologous expanded bone marrow mesenchymal stem cells: 50 cases clinical and MRI results at one year follow-up. *J Stem Cell Res Ther* **5**, 1-7 (2015).

36. Soler, R.*, et al.* Final results of a phase I-II trial using ex vivo expanded autologous Mesenchymal Stromal Cells for the treatment of osteoarthritis of the knee confirming safety and suggesting cartilage regeneration. *The Knee* **23**, 647-654 (2016).

37. Turajane, T.*, et al.* Combination of intra-articular autologous activated peripheral blood stem cells with growth factor addition/ preservation and hyaluronic acid in conjunction with arthroscopic microdrilling mesenchymal cell stimulation Improves quality of life and regenerates articular cartilage in early osteoarthritic knee disease. *J Med Assoc Thai* **96**, 580-588 (2013).

38. Centeno, C., Pitts, J., Al-Sayegh, H. & Freeman, M. Efficacy of autologous bone marrow concentrate for knee osteoarthritis with and without adipose graft. *Biomed Res Int* **2014**, 370621 (2014).

39. Jo, C.H.*, et al.* Intra-articular injection of mesenchymal stem cells for the treatment of osteoarthritis of the knee: a proof-of-concept clinical trial. *Stem Cells* **32**, 1254-1266 (2014).

40. Kim, Y.S.*, et al.* Mesenchymal stem cell implantation in osteoarthritic knees: is fibrin glue effective as a scaffold? *The American journal of sports medicine* **43**, 176-185 (2015).

41. Kim, Y.S.*, et al.* Comparative Matched-Pair Analysis of the Injection Versus Implantation of Mesenchymal Stem Cells for Knee Osteoarthritis. *The American journal of sports medicine* **43**, 2738-2746 (2015).

42. Koh, Y.G. & Choi, Y.J. Infrapatellar fat pad-derived mesenchymal stem cell therapy for knee osteoarthritis. *The Knee* **19**, 902-907 (2012).

43. Nguyen, P.D.*, et al.* Comparative Clinical Observation of Arthroscopic Microfracture in the Presence and Absence of a Stromal Vascular Fraction Injection for Osteoarthritis. *Stem Cells Transl Med* **6**, 187-195 (2017).

44. Pers, Y.M.*, et al.* Adipose Mesenchymal Stromal Cell-Based Therapy for Severe Osteoarthritis of the Knee: A Phase I Dose-Escalation Trial. *Stem Cells Transl Med* **5**, 847-856 (2016).

45. Gupta, P.K.*, et al.* Efficacy and safety of adult human bone marrow-derived, cultured, pooled, allogeneic mesenchymal stromal cells (Stempeucel(R)): preclinical and clinical trial in osteoarthritis of the knee joint. *Arthritis research & therapy* **18**, 301 (2016).

46. Koh, Y.G., Kwon, O.R., Kim, Y.S. & Choi, Y.J. Comparative outcomes of open-wedge high tibial osteotomy with platelet-rich plasma alone or in combination with mesenchymal stem cell treatment: a prospective study. *Arthroscopy* **30**, 1453-1460 (2014).

47. Lamo-Espinosa, J.M.*, et al.* Intra-articular injection of two different doses of autologous bone marrow mesenchymal stem cells versus hyaluronic acid in the treatment of knee osteoarthritis: multicenter randomized controlled clinical trial (phase I/II). *J Transl Med* **14**, 246 (2016).

48. Varma, H.S., Dadarya, B. & Vidyarthi, A. The new avenues in the management of osteo-arthritis of knee--stem cells. *Journal of the Indian Medical Association* **108**, 583-585 (2010).

49. Vega, A.*, et al.* Treatment of Knee Osteoarthritis With Allogeneic Bone Marrow Mesenchymal Stem Cells: A Randomized Controlled Trial. *Transplantation* **99**, 1681-1690 (2015).

50. Wakitani, S.*, et al.* Human autologous culture expanded bone marrow mesenchymal cell transplantation for repair of cartilage defects in osteoarthritic knees. *Osteoarthritis and cartilage* **10**, 199-206 (2002).

51. Wong, K.L.*, et al.* Injectable cultured bone marrow-derived mesenchymal stem cells in varus knees with cartilage defects undergoing high tibial osteotomy: a prospective, randomized controlled clinical trial with 2 years' follow-up. *Arthroscopy* **29**, 2020-2028 (2013).

52. Fleiss, J.L. Statistical methods for rates and proportions. (New York: Wiley, 1981).

53. Duval, S. & Tweedie, R. Trim and fill: A simple funnel-plot-based method of testing and adjusting for publication bias in meta-analysis. *Biometrics* **56**, 455-463 (2000).
